# Effects of microRNA-298 on APP and BACE1 translation differ according to cell type and 3′-UTR variation

**DOI:** 10.1038/s41598-022-05164-4

**Published:** 2022-02-23

**Authors:** Ruizhi Wang, Debomoy K. Lahiri

**Affiliations:** Laboratory of Molecular Neurogenetics‚ Departments of Psychiatry and Medical & Molecular Genetics‚ Indiana University School of Medicine‚ Indiana Alzheimer’s Disease Research Center, Stark Neuroscience Research Institute, Indianapolis, 320 West 15th Street, IN 46202 USA

**Keywords:** Biochemistry, Neuroscience, Medical research

## Abstract

Alzheimer’s disease (AD) is marked by neurofibrillary tangles and senile plaques composed of amyloid β (Aβ) peptides. However, specific contributions of different cell types to Aβ deposition remain unknown. Non-coding microRNAs (miRNA) play important roles in AD by regulating translation of major associated proteins, such as Aβ precursor protein (APP) and β-site APP-cleaving enzyme (BACE1), two key proteins associated with Aβ biogenesis. MiRNAs typically silence protein expression via binding specific sites in mRNAs’ 3′-untranslated regions (3′-UTR). MiRNAs regulate protein levels in a cell-type specific manner; however, mechanisms of the variation of miRNA activity remain unknown. We report that miR-298 treatment reduced native APP and BACE1 protein levels in an astrocytic but not in a neuron-like cell line. From miR-298’s effects on APP-3′-UTR activity and native protein levels, we infer that differences in APP 3′-UTR length could explain differential miR-298 activity. Such varied or truncated, but natural, 3′-UTR specific to a given cell type provides an opportunity to regulate native protein levels by particular miRNA. Thus, miRNA’s effect tailoring to a specific cell type, bypassing another undesired cell type with a truncated 3′-UTR would potentially advance clinically-relevant translational research.

## Introduction

Alzheimer’s disease (AD) is the leading cause of dementia globally and has no effective disease modifying therapy^1^. AD is a progressive neurodegenerative disease, pathologically characterized by extracellular deposition of amyloid plaques, intracellular neurofibrillary tangles, synaptic loss, dystrophic neurites, neuroinflammation and glia activation in vulnerable brain regions, including the hippocampus and cortex^2^. The co-existence of amyloid plaques, mainly comprising amyloid β (Aβ), and neurofibrillary tangles, mainly containing hyperphosphorylated tau protein, remains the fundamental requirement for pathological diagnosis^3^.

Aβ peptide is derived from its precursor molecule, Aβ precursor protein (APP) via sequential cleavage by β-secretase enzyme (β-site APP-cleaving enzyme, BACE1) and γ-secretase complex. Dysregulation of APP and BACE1 proteins during disease progression has long been the focus of AD research^4–6^. However, many clinical trials based upon the Aβ plaque cascade hypothesiss, trying to stop or reverse disease progression via altering Aβ levels resulted in unsatisfactory results^7^. Some renewed optimism has been fueled by aducanumab’s recent approval by the FDA as possibly the first disease-modifying anti-amyloid treatment^8^, although its unorthodox route to approval has raised scientific controversy and resignations from the FDA^9,10^. Several BACE1 inhibitor clinical trials used small pharmacological molecules including verubecestat, lanabecestat, and LY2886721 and found significant adverse effects in multiple organs besides brain^11–15^. Non-*organ*-specific overall BACE1 inhibition might account in part for the various adverse effects^11^. Thus, regulation of APP and BACE1 in an organ or cell type specific manner is very important, a goal that can be achieved by microRNA (miRNA) regulation of mRNA translation.

AD transcriptomes shows progressive changes in hippocampal functions, epigenetics and miRNA regulation^16^. AD is marked by cerebrovascular deposition of Aβ peptides; however, specific contributions of different tissue and cell types to Aβ deposition remain unknown. We try to bridge this knowledge gap by studying the role of specific miRNAs that play key roles in AD by regulating key proteins involved.

MiRNAs are short non-coding RNAs with mature length around 22 nucleotides (nt). They function as important regulators of mRNA translation. MiRNAs within the RISC complex generally inhibit target mRNA expression by either transcription inhibition or mRNA degradation. MiRNA binding to target mRNA 3′-UTR usually depends on its seed sequence located at 2–8 nucleotide from the miRNA 5′ end. Seed sequence base pairing with target mRNA 3′-UTR is exactly complementary though base pairing on other sites may not be perfect^17,18^.

Regulation of APP and BACE1 mRNAs by miRNAs has become the primary focus in the field. APP mRNA is regulated by multiple miRNAs including miR-20b, miR-101, miR-153, miR-31, miR-346, miR-106a, and miR-520c^19–26^. BACE1 mRNA is regulated by miR-124, miR-339-5p, and miR-29c^27–29^. Notably, miR-298 reduced both APP and BACE1 levels^30,31^.

In this context, we have recently reported^31^ that miR-298 targets APP and BACE1 mRNA 3′-UTRs. In addition, miR-298 treatment reduced two major potentially toxic forms of Aβ peptides (Aβ 1–40 and Aβ 1–42) in primary human mixed cell cultures derived from human fetal brain samples, presumably by reducing APP and BACE1 proteins. Surprisingly, miR-298 also reduced a specific isoform of tau protein. Furthermore, miR-298 levels and single nucleotide polymorphism (SNPs) associated with AD progression. However, specificity of miR-298 functions regarding cell types including neurons, microglia and astrocytes has been poorly studied.

Several miRNA profiling studies have demonstrated that miRNA levels vary greatly in different cells, tissues and organs^32^. But differences in miRNA function and targeting in cell and tissue specific manners have not been well studied, especially mechanisms. MiRNA and mRNA transcript interaction can be extensively regulated via multiple potential mechanisms, which can be leveraged to clinical translational advantages. Alternative polyadenylation could shorten the length of mRNA 3′-UTRs which can delete miRNA binding sites. Further, the presence of a SNP within or around miRNA binding sites might alter the folding energy and hence binding affinity. Moreover, endogenous miRNA levels vary greatly among various cell types. The presence of high level of miRNAs could saturate the target. This is distinctly likely because miRNA-mRNA interaction follows Michaelis–Menten kinetics^33^. In addition, mRNA binding proteins that interfere with miRNA binding can also be expressed in a cell-type specific manner.

In the present study, we attempted to address miRNA’s activity at the cellular level. We posit that, although miRNA binds a specific seed sequence in the 3′-UTR of a target mRNA, variation in UTR length that results in omitting seed sequences could prevent a miRNA’s binding and, thus, its activity in a particular human cell line derived from a specific cell type. The “natural” 3′- UTR could be as long as the database-reported “full length” (e.g., 1133 nt for APP-3′-UTR) in a particular cell type or truncated (undefined) in another. We have considered several methods to determine the exact length and sequences of target mRNA 3′-UTR to account for differential miRNA activity. For example, ‘Rapid amplification of cDNA ends’ (RACE) is a common technique to obtain the sequence of an RNA transcript found within a cell. However, sequence information *alone* would not be sufficient to understand the activity of miRNA and hence its *function* in regulating cellular protein.

Herein we describe “miRNA-associated native protein expression” or “miRnape” by miRNA transfection and  then cellular protein expression assays (protein and also mRNA levels). We present data that miRnape assay (see below) can determine a *natural* “UTR limit” for a miRNA’s function in a cell type or cell line in our case. Briefly, we selected several miRNAs that are located at different locations within APP-3 ‘-UTR. We checked the function of each miRNA on APP-3′-UTR activity using the full-length UTR and dual reporter assay. Since miRNAs bind to target sequence on mRNA 3′-UTR and inhibit protein, we checked protein expression too. In short, by selecting a range of miRNAs with different binding sites located at different positions on the same target mRNA 3′-UTR, we could map the biologically active 3′-UTR. The miRnape technique would be useful in understanding a miRNA’s cellular function.

We show the effects of miR-298 in different cell lines as well as primary neural stem cells (NSCs). MiR-298 reduces APP and BACE1 in one type of human astrocytes U373 but not in human differentiated neuroblastoma or microglia cells. Our results suggest that miR-298 regulates its targets in a cell type specific manner by leveraging full-length or truncated 3′-UTRmRNA.

## Results

### Bioinformatic analyses of APP- and BACE1 3′-UTR reveal potential miRNA binding sites and polyadenylation sites

**Figure 1:**
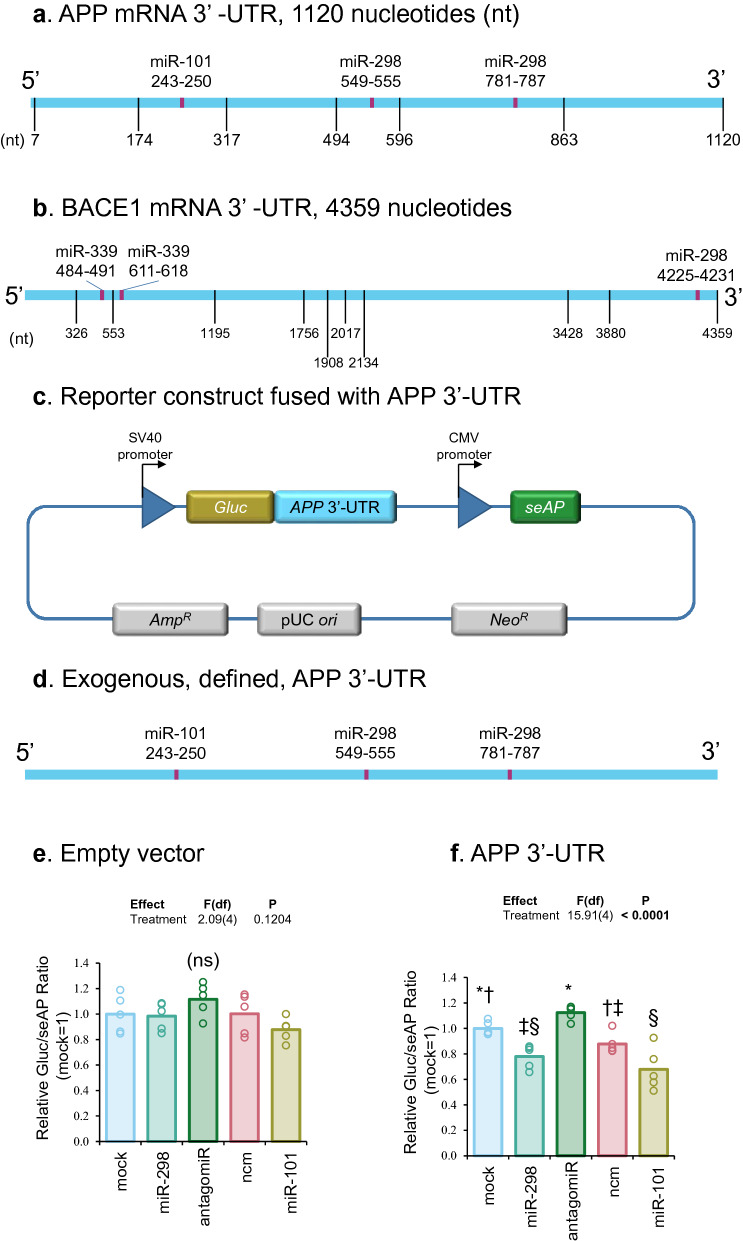
Schematic diagram showing APP and BACE1 mRNA 3′-UTR and effects of miR-298 on APP 3′-UTR activity in human differentiated neuroblastoma cells. (**a**,**b**) Scale drawings (not to same scale) of APP and BACE1 mRNA 3′-UTR separately along with potential polyadenylation sites and miRNA binding sites. Positions of miR-101, miR-298 and miR-339 seed sequence binding sites are shown. Potential polyadenylation sites are indicated by vertical lines and miRNA binding sites are shown as red boxes. (**c**) APP mRNA 3′-UTR was inserted into pEZX-MT05 plasmid downstream of secreted Gaussia luciferase (GLuc) gene. Another reporter gene in the plasmid, secreted Alkaline Phophastase (seAP), is independently transcribed and used as an internal control. (**d**) Scale drawing of exogenous APP mRNA full length 3′-UTR with miR-101 and miR-298 binding sites shown. (**e**,**f**). Plasmid pEZX-MT05 and miRNAs were co-transfected in differentiated neuronal cells, and thereafter processed as described in the ‘Material and methods’. Different treatment groups are as indicated in the figure. Briefly, cells were harvested and conditioned media were split into two parts to do separately GLuc and seAP luminescence assays. The GLuc/seAP ratio represents the target 3′-UTR activity, and data were analyzed. MiR-298 and miR-101 significantly reduced APP 3′-UTR activity but not empty vector versus mock transfection. Results of pairwise comparison of all treatments is shown by symbols. Treatments sharing a symbol did not significantly differ (p < 0.05). "(ns)" indicates no significant pairwise differences.

We consulted online bioinformatics tools to find potential polyadenylation sites within APP and BACE1 mRNA 3′-UTRs^34^. Alternative polyadenylation could shorten 3′-UTR lengths, thus interfering with or even preventing miRNAs from binding to their target sequences (Fig. 1a,b). We obtained potential miR-101, miR-298 and miR-339-5p binding sites on APP and BACE1 3′-UTR from several bioinformatics prediction tools including TargetScan^35^, StarMir^36^, miRDB^37^, miRmap^38^, and RNA22^39^. Several of these sites were experimentally validated in our lab and published in our previous works^25,29,31^. The seed sequence binding site of miR-101 (242–249 nt counting from the beginning of the 3′-UTR) on APP 3′-UTR is upstream of miR-298 binding sites (549–555 nt, and 780–786 nt). In addition to APP, we also studied BACE1 regulation. The seed sequences of binding sites of miR-339-5p (484–490, and 610–617 nt) on BACE1 3′-UTR are also upstream of miR-298 binding sites (4225–4231 nt). Potential alternative polyadenylation could truncate the APP- or BACE1 3′-UTRs, deleting miR-298 binding sites.

### MiR-298 reduced exogenous, full-length, defined APP 3′-UTR activity

Full length, 1133 nt, well defined APP 3′UTR was cloned into a reporter vector and co-transfected with miRNAs into differentiated neuroblastoma SK-N-SH cells. (Fig. 1c,d) MiR-101 and miR-298 binding sites are shown in Fig. 1d. Separate treatment of miR-298 and miR-101 significantly reduced APP 3′-UTR activities as compared to APP 3′-UTR constructs alone and APP 3′-UTR with miR-298 inhibitors. (Fig. 1e,f) They did not significantly alter the activities of empty vector. Both miR-101 and miR-298 were able to bind exogenous full-length APP 3′-UTR and reduced its activity.

### MiR-298 administration reduced APP and BACE1 mRNA in astrocytes but not in differentiated neuroblastoma cells

**Figure 2:**
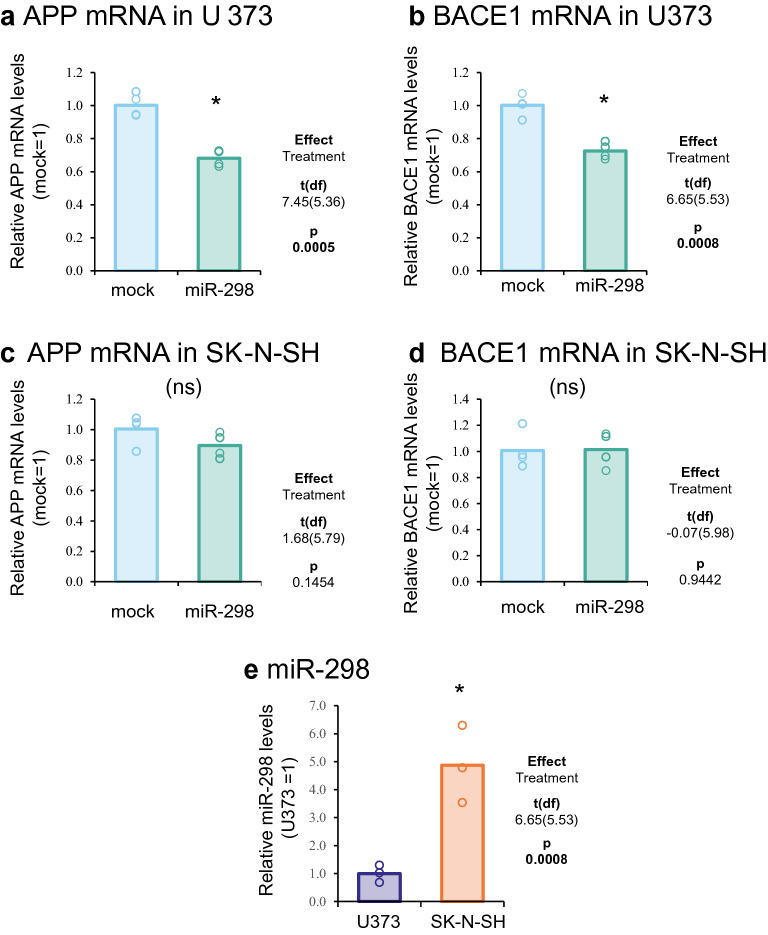
Effects of miR-298 treatment on APP and BACE1 mRNA in astrocytes and differentiated neuroblastoma cells. (**a**, **b**) Astrocytes were transfected with miR-298 for 3 days. RNA was harvested for reverse transcription and following quantification by real-time qPCR. APP and BACE1 mRNA showed significant reduction when transfected with miR-298. (**c**,**d**) Real-time qPCR analysis in differentiated neuroblastoma cells transfected with miR-298. (**e**) Quantification of endogenous miR-298 levels in astrocytes U373 and NBRA differentiated SK-N-SH. MiR-298 levels were normalized with the geometric means of miR-16, RNU48 and RNU6B. Significant pairwise difference indicated by "*".

MiR-298 significantly reduced (~ 30%) APP and BACE1 mRNA levels in astrocytes (U373) (Fig. 2a,b) but not in differentiated neuroblastoma cells (SK-N-SH) (Fig. 2c,d). It indicates that miR-298 associated with degrading target mRNA transcripts in astrocytes. Endogenous miR-298 level was significantly higher in differentiated neuroblastoma cells than in astrocytes (Fig. 2e). The miR-298 level was quantified by normalization with several other small non-coding RNA miR-16, RNU48, and RNU6B, validated and applied in various studies as reference small RNAs^40–42^.

### MiR-298 significantly reduced endogenous native APP and BACE1 protein levels in human astrocyte cells

**Figure 3:**
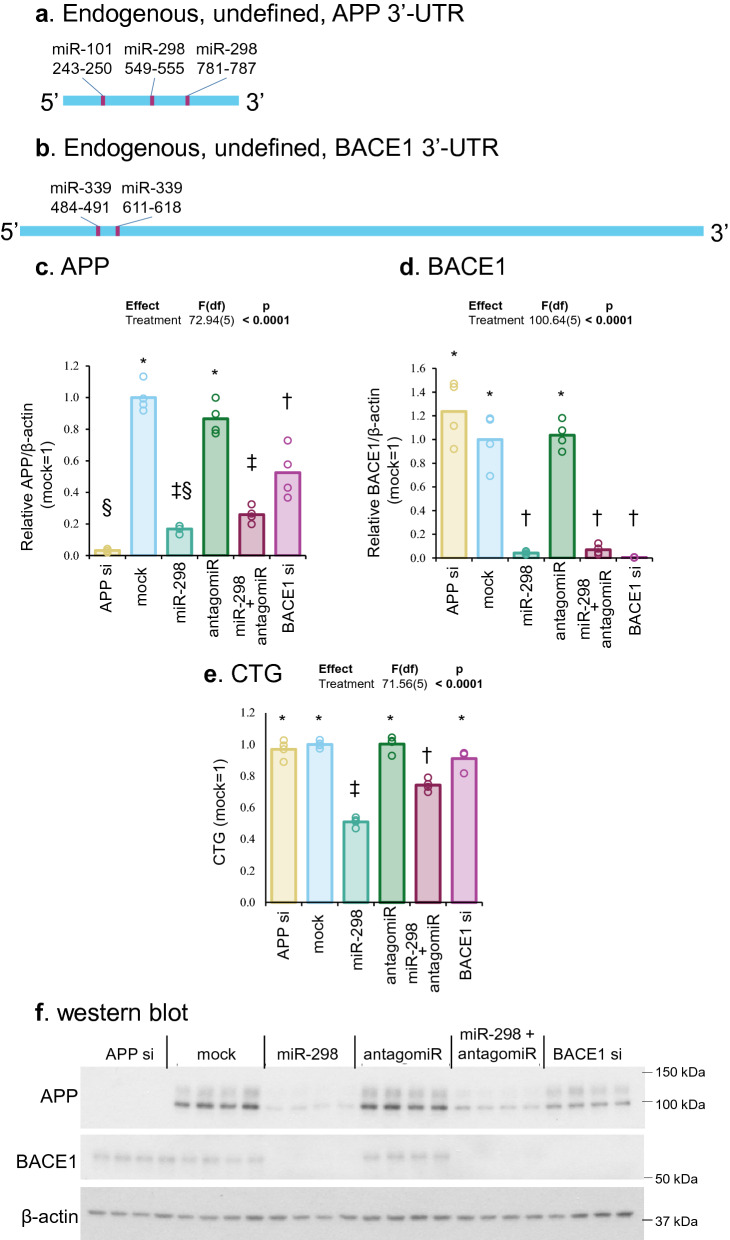
Effects of miR-298 on endogenous APP and BACE1 levels in human astrocyte cells. (**a**,**b**) Scale drawings of endogenous APP and BACE1 mRNA undefined 3′-UTR with miR-298 binding sites. With full-length 3′-UTR, where miRNAs can bind, miR-298 can reduce APP and BACE1 expression levels. (**c**,**d**) Densitometric analysis of APP and BACE1 levels. Each symbol represents a different statistical group. (**e**) Cell viability determined by CTG assay. (**f**) Western blotting of APP, BACE1 and β-actin. Results of pairwise comparison of all treatments is shown by symbols. Treatments sharing a symbol did not significantly differ (p < 0.05). "(ns)" indicates no significant pairwise differences.

First, we mapped appropriate miRNA binding sites (e.g., miR-339 and miR-298) that should be present in endogenous, native, undefined APP and BACE1 3′-UTRs (Fig. 3a,b). Notably, miR-298 overexpression significantly reduced (~ 85–95%) levels of APP and BACE1 protein in human astrocyte cells *vs.* mock transfection. When miR-298 and antagomiR (anti-miR-298) were co-transfected, protein reduction was reversed, though partially. The antagomiR alone did not alter levels of APP or BACE1 (Fig. 3c–e), suggesting that interaction between the antagomiR with endogenous miR-298 is partial. Alternatively, endogenous miR-298 levels were too low that there was no functional binding on its target mRNAs and that further inhibition by the antagomiR could not significantly alter target protein levels. Besides reduced APP and BACE1 protein levels, cell viability was also significantly reduced by miR-298 in astrocytes (Fig. 3f).

### MiR-298 did not reduce endogenous APP protein in differentiated neuroblastoma cells

**Figure 4:**
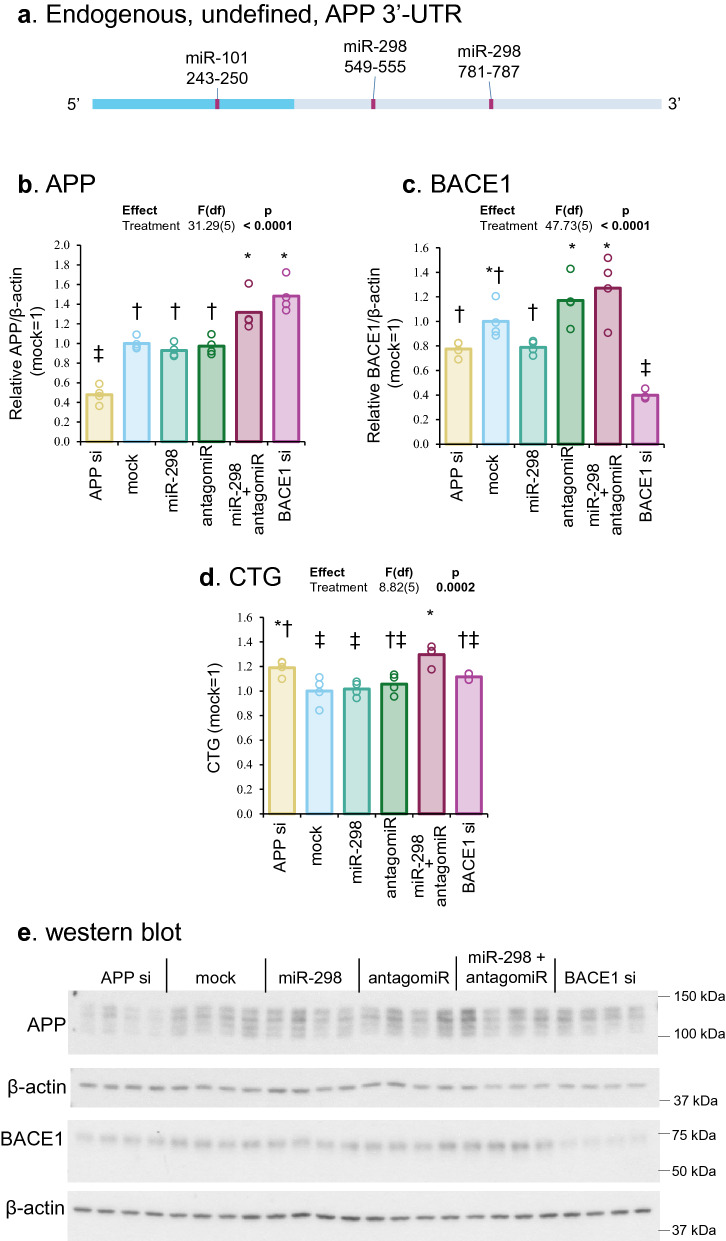
Effects of miR-298 on endogenous APP levels in differentiated neuroblastoma cells. (**a**) Scale drawing of endogenous APP mRNA undefined 3′-UTR with miR-101 and miR-298 binding sites. Dashed line represents potential truncation or alternation of APP 3′-UTR, where miR-298 binding sites could be absent. (**b**,**c**) Densitometric analysis of the blot. (**d**) Cell viability analysis by CTG assay. (**e**) Western blotting of APP, BACE1 and β-actin protein. Results of pairwise comparison of all treatments is shown by symbols. Treatments sharing a symbol did not significantly differ (p < 0.05). "(ns)" indicates no significant pairwise differences.

MiR-101 and miR-298 binding sites on APP 3′-UTR were mapped. Potential 3′-UTR modifications could prevent miR-298 from binding (Fig. 4a). MiR-298 treatment did not significantly reduced APP protein versus mock transfection (Fig. 4b,c); and it was not significantly different from miR-298 inhibitor (antagomiR) treatment. Also, we noted different profiles of APP bands between these cell types, which could be due to splicing variants, and posttranslational modifications (Figs. 3, 4). MiR-298 treatment did not alter BACE1 protein levels (Fig. 4d). To determine whether transfection of a small RNA is hindered in differentiated NB cells, we independently transfected cells with APP and BACE1 siRNA, which resulted in significant reduction of APP and BACE1 levels, respectively, suggesting that siRNA transfection was unhindered in these cells (Fig. 4e). MiR-298 treatment did not change neuronal cell viability in contrast to the significant reduction in U373, where both APP and BACE1 levels were significantly reduced (Fig. 3e).

### MiR-339-5p but not miR-298 changed endogenous BACE1 protein levels in differentiated neuroblastoma cells

**Figure 5:**
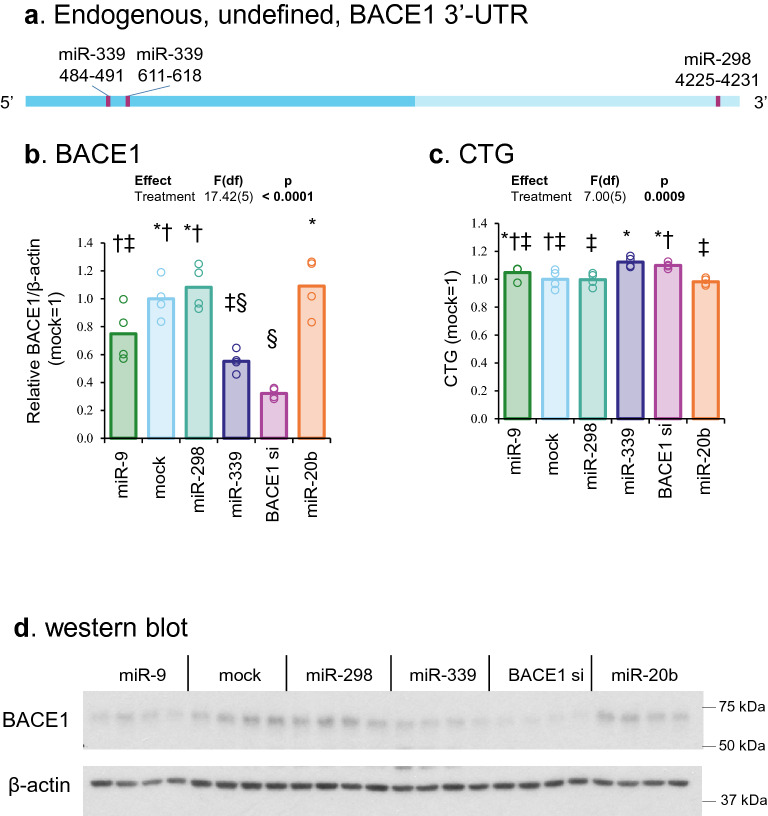
Effects of miR-298 and miR-339 on endogenous BACE1 levels in differentiated neuroblastoma cells. (**a**) Scale drawing of endogenous BACE1 mRNA undefined 3′-UTR with miR-339 and miR-298 binding sites. Dash line represents potential truncation or alternation of BACE1 3′-UTR. (**b**) Densitometric analysis. (**c**) CTG assay. Each letter represents a different group. (**d**) Western blotting of BACE1 and β-actin. Results of pairwise comparison of all treatments is shown by symbols. Treatments sharing a symbol did not significantly differ (p < 0.05). "(ns)" indicates no significant pairwise differences.

We mapped miRNA binding sites (e.g., miR-339-5p and miR-298) in BACE1 3′-UTR that should either be present or absent in endogenous native BACE1mRNA depending on the cell type (Fig. 5a). Like in APP3′-UTR described before, specific potential alternation or variation could interfere with miR-298 binding. (Fig. 5a) Notably, miR-339-5p transfection but not miR-298 significantly reduced native BACE1 protein (Fig. 5b, c). Likewise, cell viability was not changed by miR-298. (Fig. 5d).

### MiR-298 treatment in neural stem cells did not alter APP or BACE1 protein levels

**Figure 6:**
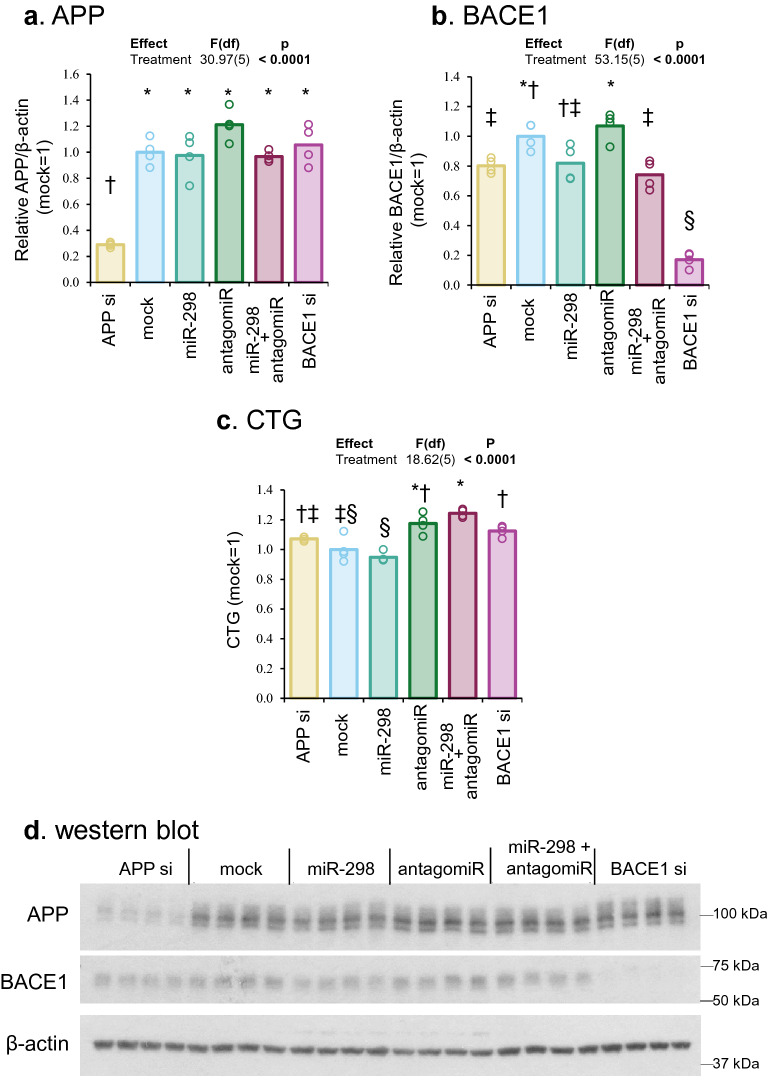
Effects of miR-298 on endogenous APP and BACE1 levels in neural stem cells derived from iPSC. (**a**,**b**). Densitometric analysis. (**c**) CTG assay. Each symbol represents a different group. (**d**) Western blotting of APP, BACE1, and β-actin. Results of pairwise comparison of all treatments is shown by symbols. Treatments sharing a symbol did not significantly differ (p < 0.05). "(ns)" indicates no significant pairwise differences.

Since U373 and SK-N-SH are both cancer cell lines, some of their cancer features such as genome alteration may interfere with interpretation. We applied neural stem cells (NSC) derived from footprint-free and karyotype normal human induced pluripotent stem cells (iPSCs) instead. Notably, miR-298 treatment did not change APP or BACE1 protein levels (Fig. 6a–c), while APP and BACE1 siRNA successfully reduced target protein expression, serving as positive controls. Cell viability was also not changed by miR-298 compared to mock transfection (Fig. 6d).

### MiR-298 treatment’s effect on APP and BACE1 proteins depends on “intrinsic properties” of each cell type

We recognize that each cell type has not only potentially specific 3 ‘-UTRs for mRNAs, either full-length or truncated, but also other cellular ‘factors’ intrinsic to each cell type. Would cell-type specific factors, such as secreted proteins, cytokines, and small molecules explain differential miR-298 results (Figs. 4, 5, 6)? Some proteins are secreted into cell culture media. Since cells are allowed to grow 72 h prior to harvest, could such secreted products influence the outcome of miR-298 in a cell-type manner? We reasoned that proteins and other products, usually secreted into the conditioned medium, could be a good proxy for each cell type. For this, we divided the experiment in the following way.

**Figure 7:**
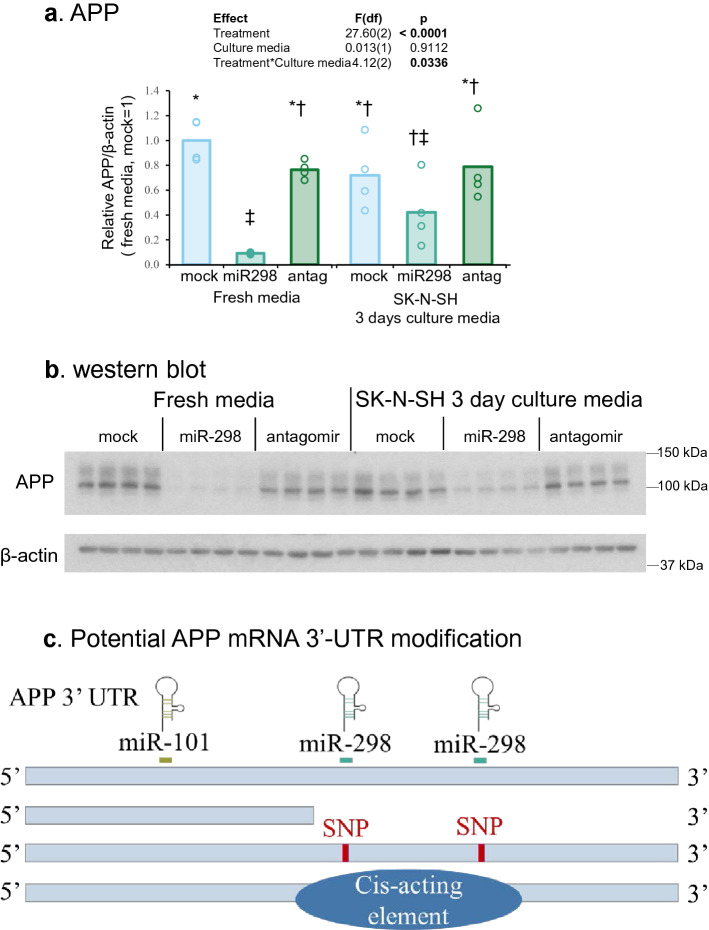
miR-298 treatment’s effect on APP and BACE1 proteins depends on the intrinsic property of each cell type and possible scenarios. (**a**) Western blot of APP and β-actin proteins. (**b**) Densitometry analysis. Each symbol represents a different group. (**c**) Schematic display of potential ways of regulation on APP mRNA 3′-UTR. The miR-298 binding sites on APP 3′-UTR are located downstream of miR-101. Several scenarios that lead to truncated 3′-UTR and could interfere miR-298 binding include alternative polyadenylation sites, presence of SNPs, mutations in miR-298 binding sites and cis-acting elements. Results of pairwise comparison of all treatments is shown by symbols. Treatments sharing a symbol did not significantly differ (p < 0.05). "(ns)" indicates no significant pairwise differences.

One set of astrocytes (U373) were transfected with miR-298 and cultured in fresh opti-MEM media for 3 days. The second set of astrocytes were similarly transfected with miR-298 but cultured in opti-MEM media conditioned by differentiated neuroblastoma cells (SK-N-SH) for 3 days. Such media exchange, from ‘negative-effect’ SK-N-SH cells to U373 cells to, did not significantly change effects observed in U373 cells (Fig. 7a,b) Therefore, miR-298’s effect remained the same irrespective of post-transfection media change in astrocytes. Thus we attribute it to intrinsic property of the cell type used herein. We also performed the reverse (cell type) experiment. The first batch of neuroblastoma (SK-N-SH) cells were transfected with miR-298 and cultured in fresh opti-MEM media. The second set of SK-N-SH cultures were transfected with miR-298 but cultured in opti-MEM media conditioned by U373 cells for 3 days. As before, such media exchange did not significantly change outcomes. (Supplementary Fig. 1a,b) In differentiated neuroblastoma, miR-298’s “negative-effect” was not altered in the second set even though the cells received media from the “positive” U373 cells at the post-transfection stage.

## Discussion

The central nervous system (CNS) is a remarkably complex organ system, requiring an equally complex network of molecular pathways controlling the multitude of diverse cellular activities. Gene expression is a critical node at which regulatory control of molecular networks is implemented. As such, elucidating the various mechanisms employed in the physiological regulation of gene expression in the CNS is important both for establishing a reference for comparison to the diseased state and for expanding the set of validated drug targets available for disease intervention. MicroRNAs (miRNAs) are an abundant class of small RNA that mediates potent inhibitory effects on global gene expression. Recent advances have been made in methods employed to study the contribution of these miRNAs to gene expression^43^. Here we present a methodological workflow from the perspective of an investigator studying the physiological regulation of a gene of interest. We discuss methods for identifying putative miRNA target sites in a transcript of interest, strategies for validating predicted target sites, assays for detecting miRNA expression, and approaches for disrupting endogenous miRNA function. We consider both advantages and limitations, highlighting certain caveats that inform the suitability of a given method for a specific application. Through careful implementation of the appropriate methodologies discussed herein, we hope that important discoveries related to miRNA participation in CNS physiology and dysfunction are on the horizon.

Our lab has studied the mechanisms of cell-type-specific regulation of APP, APOE, and BACE1 genes, focusing primarily on promoters and 5′-flanking regions^44–49^. The present novel findings of miRNA’s role on protein expression, focusing on the 3′-UTR of APP and BACE1, add significantly to our previous studies on gene regulation. Herein, we posit that although miRNA binds a specific seed sequence in target mRNA 3′- UTR, the variation in UTR length might prevent a miRNA’s binding and, thus, affects its activity in a particular cell type. In this study, we demonstrated that miR-298 significantly reduced APP and BACE1 levels in human astrocytes at both protein and mRNA levels. But miR-298 did not alter APP or BACE1 levels in other human cell lines. Surprisingly, the effects of one miRNA could be so diversified and even contrasting in different cells. The cell-type-specific regulation by miRNAs has not been well studied. Few cases have been reported, but the exact mechanism of the phenomenon remains unclear^50^.

We embarked on this study to answer a fundamental question. It is known that miRNA regulates protein levels in a cell-type specific manner but how miRNA functions differently in various cell types remains elusive. We postulated that although miRNA binds a specific seed sequence in the 3′-UTR of a target mRNA, the variation in UTR length could prevent a miRNA’s binding and thus affect its activity in a particular cell type. We reasoned that the natural 3′-UTR could be a full length (e.g., 1.12 nt for APP-3′-UTR and 4.36 nt for BACE1-3′-UTR) in a particular cell type or truncated (undefined) in another. The main objective of our work was to address this issue as far as miRNA’s function is concerned.

We deliberated other methods to determine the exact sequences of target mRNA 3′-UTR in order to account for differential miRNA activity. For example, the RACE technique is generally used to obtain the full-length sequence of an RNA transcript found within a cell. RACE produces a cDNA copy of the RNA sequence of interest, generated through reverse transcription, followed by PCR amplification of the cDNA copies. It involves using one common primer that takes advantage of mRNA transcript poly (A) tail and another customized primer. In essence, the PCR amplification is one-sided PCR with single-sided specificity. Also, such a sequence is not sufficient to understand the activity of miRNA and hence its function in regulating cellular protein. While it is true that 3′-UTR-mRNA sequence alone will not elucidate every single tiny aspect of mRNA-miRNA interaction, the sequence information will determine if a cell tends to favor truncated 3′-UTR sequences. In this context, the miRnape approach emphasizes the functional aspect of mRNA-miRNA interaction regarding a miRNA's effects on UTR-based reporter activity and native protein expression levels.

Instead of RACE, we utilized a miRnape to determine a natural “UTR stop” in a cell type. In essence, we selected several miRNAs that are located at different locations within APP 3′-UTR or BACE1 3′-UTR. We checked the function of each miRNA on UTR activity using the full-length 3'-UTR and by dual reporter assay. Then we checked the function of each miRNA on native protein levels. Transfection results of known miRNA on native protein expression would determine whether UTR is fully active (positive results) or truncated (negative results) in a particular cell type. We then matched the results by doing transfection experiment with a known full-length UTR. Using a range of miRNAs with binding sites spread across the same target mRNA transcript 3′-UTR, we could map the functional miRNA’s site within 3′-UTRmRNA. Our miRnape approach is useful in understanding cellular function of a miRNA.

To explain our work, we also propose several potential hypotheses and additional explanations. For example, 3′-UTR can either be truncated, mutated, or bound by some cis-acting elements as shown in Fig. 7c.

Scenario 1: Endogenous miR-298 levels vary in various cells. It is possible that in some cells miR-298 levels are sufficiently high that its targets are already saturated. Additional exogenous miR-298 mimic transfected into cells could not further reduce endogenous APP and BACE1 levels. However, this hypothesis is not favored in our case based on the two pieces of evidence. First, endogenous miR-298 levels vary little compared to exogenous miR-298 transfection. An about fivefold elevation in differentiated neuroblastoma cells may not likely to saturate its target. Second, even if endogenous miR-298 had already saturated to its targets, exogenous miR-298 inhibitors might disengage that interaction and significantly increase APP and BACE1 levels, which is not the case. Our challenge is to recognize that mRNA: microRNA interaction kinetics are not linear.

Scenario 2: Alternative polyadenylation sites or SNPs are present within mRNA 3′-UTR targeted by miR-298. Our results suggest that endogenous APP protein was reduced only in U373 but not HMC3, HeLa or differentiated neuroblastoma cells, and that a full-length APP 3′-UTR activity reporter activity was reduced in differentiated neuroblastoma cells when co-transfected with miR-298 mimics. These results suggest that endogenous APP 3′-UTRs may not be identical in all cell types. There are two possibilities, either alternative polyadenylation sites make the APP 3′-UTR shorter in some cells or the APP 3′-UTR has cell culture-originating SNPs in miR-298 binding region that reduces miR-298 binding affinity. However, any SNPs reported in the NCBI SNP database are located within or close to miR-101, miR-298, and miR-339 seed sequence binding sites on APP and BACE1 mRNA 3′-UTR, are of very low frequency (< 0.03%). The presence of such a low frequency of related SNPs would make it unlikely that they explain the miRNA effect differences observed by these rare SNPs, some of which are listed in Table 1. However, that does not prove that these specific cells do not have SNPs that have not been reported yet,

**Table 1:** SNPs located within or close to miRNA seed sequence binding sites*

miRNA	Target	SNP	Frequency
miR-101	APP 3′-UTR	rs1568997318	0.000007
miR-101	APP 3′-UTR	rs1439565783	0.000057
miR-101	APP 3′-UTR	rs1260407227	0.000007
miR-101	APP 3′-UTR	rs2036975815	0.000007
miR-298	APP 3′-UTR	rs1479517600	0.000043
miR-298	APP 3′-UTR	rs191651536	0.0002
miR-298	APP 3′-UTR	rs2036942563	0.000004
miR-298	APP 3′-UTR	rs2036942344	0.000004
miR-298	APP 3′-UTR	rs1216676820	0.000007
miR-339	BACE1 3′-UTR	rs570503330	0.000091
miR-339	BACE1 3′-UTR	rs2034340128	0.000004
miR-339	BACE1 3′-UTR	rs2034340051	0.00006
miR-339	BACE1 3′-UTR	rs1159500293	0.000004
miR-339	BACE1 3′-UTR	rs1053615732	0.000008
miR-339	BACE1 3′-UTR	rs755195807	0.00015
miR-298	BACE1 3′-UTR	rs910101318	0.000004
miR-298	BACE1 3′-UTR	rs1037052324	0.000004
miR-298	BACE1 3′-UTR	rs922820472	0.000019

*SNP information was obtained from dbSNP of National Center for Biotechnology Information.

https://www.ncbi.nlm.nih.gov/snp/rs1568997318?vertical_tab=true.

Scenario 3: Some cis-acting elements bind to APP and BACE1 mRNA 3′-UTR and may prevent miR-298 from binding. For example, TGF-β treatment increased APP mRNA levels via an unidentified 68 kDa protein binding to APP 3'-UTR^51^. HuD promoted both APP and BACE1 mRNA stability by interacting with their mRNA 3′-UTR^52^. Other proteins, such as hnRNP C and fragile X mental retardation protein (FMRP) compete with each other for APP 3′-UTR binding^53^. Their binding on 3′-UTR could potentially affect miRNA binding. Further, the hypothesis that cell-type specific RNA binding proteins can bind to target mRNA and hence mask further miRNA binding was raised and described in detail^54^. MiRNA machinery in different cells could work differently, leading to different effects of one miRNA in various cell types. Even miR-298 and its target mRNA 3′-UTR are identical, and the results might differ in multiple cells. In the case of exogenous APP 3′-UTR transfection experiments, APP 3′-UTR are over-expressed transiently into the cells compared to endogenous APP mRNA. The abundance of exogenous APP 3′-UTR may make endogenous cis-acting elements unavailable, which bind native APP mRNA. In other words, exogenous APP 3′-UTR might not be as tightly controlled as native APP mRNA.

In the future, sequences of native APP and BACE1 mRNA3′-UTRs for various cell types would be important to check UTR lengths and differences in polyadenylation. Likewise, sequences of genomic DNA from different cell cultures containing APP and BACE1 genes would confirm whether SNPs near the miR-298 binding site might interfere with the binding. Further, although we have shown results from primary iPSC-derived NSCs, transfection of miR-298 mimics in primary or iPSC derived astrocytes and microglia and neurons derived from same patient would be an important model. The role of miR-298 in these cells would have critical biological significance.

In addition to Aβ generation, APP also plays critical roles in multiple biological and pathological processes, including synaptic pruning, inflammation, iron regulation and mild traumatic brain injury (mTBI)^55–57^. Indeed, insufficient pruning could be a potential cause of autism spectrum disorder^58,59^.

Likewise, BACE1 serves more than an APP cleaving enzyme and a pathogenic role in AD. BACE1, an aspartic protease, has many other native substrates in the brain, such as neuregulin^60^, seizure protein 6^61^ and sodium gated voltage channel β2 (Navβ2)^62^, which are important for neuron function and biology. Other BACE1 substrates are involved in cell signaling and immunity including Golgi localized membrane-bound α-2,6–sialyltransferase (ST6Gal I)^63^, interleukin-1 type II receptor (IL1R2)^64^, P–selectin glycoprotein ligand–1 (PSGL-1)^65^ and low density lipoprotein receptor–related protein (LRP)^66^. By reducing BACE1 levels in astrocytes instead of neurons, we could possibly inhibit pathological Aβ production while saving the physiological functions BACE1 has in neurons.

We have also considered the translational implication of our work based on miRnape. We suggest that truncated but *natural*, 3′-UTRs found provides an avenue to regulate native protein levels by a particular miRNA in a cell type-specific manner. In short, while a traditional chemical or drug would have access to any cells, miRNA’s (e.g., miR-298) biological effects can be tailored to a specific cell type (e.g., astrocytic line) over another undesired cell type (e.g., neurons) with a 3′-UTR truncated enough to lack a miRNA binding site. Likewise, other miRNAs and their target 3′-UTRs can be tested by the miRnape method. Future work is also warranted to study different scenarios and potential outcomes, as described above, to achieve optimal miRNA activity in regulating native protein expression.

## Materials and methods

### Cell culture

Different cell lines, such as human glioblastoma (U373), neuroblastoma cells (SK-N-SH), microglia (HMC3) and HeLa cells, were obtained from ATCC. Cells were grown in Eagle’s modified minimum essential media (EMEM) containing 10% FBS and penicillin/streptomycin solution at 37 °C in 5% CO2 humid incubators as described^67^. Neuronal cultures were achieved by differentiating SK-N-SH cells with 10 µM all-trans retinoic acid (ATRA, Sigma) for 7 days in 2% FBS maintenance media. Neuronal morphology with neurites outgrowth and synaptic proteins expression including SNAP-25, synaptophysin and PSD95 were confirmed by microscope imaging or western blotting respectively. Neural stem cells were originated from blood cells of footprint-free and karyotype normal male individual’s .iPS cells (Applied Stemcell Inc.).

### Identification of potential polyadenylation sites on APP and BACE1 3′-UTR and miR-101, miR-298 and miR-339 binding sites

Multiple bioinformatics tools were consulted, which predict miRNA binding sites on target mRNA 3′-UTR via different algorithms^35–39^. Potential poly (A) sites were obtained from miRIAD database^34^.

### Cloning of APP 3′-UTR and BACE1 3′-UTR into a dual reporter vector

We performed the cloning of APP 3′-UTR (gene accession NM_000484.3) in the following way. APP 3′-UTR (1,120 nt) was cloned into a dual luciferase assay reporter vector pEZX-MT05 (GeneCopoeia), downstream of Guassia luciferase (GLuc) gene, which is driven by SV40 promoter. A synthetic poly (A) tail follows APP 3′-UTR sequence. The pEZX-MT05 plasmid is 8,600 nt long containing a separately transcribed gene, Secreted Alkaline Phophastase (seAP) luciferase gene, with its own CMV promoter and poly(A) tail served as an internal control. Likewise, we cloned BACE1 3′-UTR (4,359 nt) (gene accession NM_012104.4) into a dual luciferase assay reporter vector pEZX-MT05 as described above. The ratio of GLuc versus seAP luciferase readings represents target 3′-UTR activity.

### Reporter assay to measure UTR activity

GLuc and seAP luciferase activities were separately measured following the manufacturer’s instructions (GeneCopoeia). Briefly, cell transfection media supernatants were collected and mixed with GLuc or seAP substrates. The luciferase intensities of mixes were then measured by a Veritas microplate luminometer (Turner Biosystems).

### Transfection

Transfections were performed when cells reached around 80% confluence. Culture media were replaced with Opti-MEM media with 1% FBS and antibiotics were omitted from transfection media. For miRNAs and siRNAs transfection, Lipofectamine RNAiMax was applied 2 µl per well in a 24 well plate format. Then 75 nM miRNA or 50 nM siRNA were premixed with RNAiMax according to the protocol. The newly formed RNA mimics-transfection complexes were added into each well and kept for 72 h or otherwise indicated in figure legends. For co-transfection of miRNAs with plasmid, cells were treated with Lipofectamine RNAiMax and reporter vector alone or along with 75 nM miRNAs. Cell culture media were harvested for luciferase assay after 72 h.

### Lysis of cells

After washing with PBS, cells were lysed on-plate with vigorous shaking using 100 µl RIPA buffer containing 1 × Halt Protease Inhibitor Cocktail (Thermo Scientific). Protein concentration was determined by BCA (ThermoFisher Scientific) assay according to the manufacturer’s instructions, and then Laemmli sample buffer (LSB) was added to each tube of lysate. Lysate and LSB mixes were boiled for 10 min and cooled down on ice or kept in freezers for further studies.

### SDS–polyacrylamide gel electrophoresis (SDS–PAGE) and western blotting

An equal amount of protein lysate was loaded onto 26 lane BisTris XT denaturing 4–12% polyacrylamide gels and run with XT MOPS or XT MES buffer at 200 V for 1 h. Proteins were separated with SDS-PAGE and then transferred overnight onto PVDF membranes. Membranes were stained with 0.1% Ponceau S solution to confirm transfer success. After three times of washing using TBS with 0.05% Tween 20 (TBST), membranes were incubated with 5% nonfat milk in TBST for 1 h at room temperature. Primary antibodies were incubated at either room temperature for 3 h or 4 °C overnight. Primary antibodies used in this study are: APP (Millipore MAB348), BACE1 (Cell Signaling D10E5) and β-actin (Sigma A-5441). Either goat anti-rabbit or mouse secondary antibodies was applied for 1 h at room temperature. Protein bands were visualized using ECL and autoradiography. Films were scanned for densitometry analysis.

### RNA isolation and real-time quantitative PCR

Total RNA was isolated with mirVana miRNA isolation kit (ThermoFisher Scientific) following the manufacturer’s protocol. RNA concentration was assessed by Nanodrop instrument (ThermoFisher Scientific). Equal amount of RNA per sample was reverse transcribed with High Capacity RNA-to-cDNA kit (Applied Biosystems) for mRNA quantification. For miRNA quantification, total RNA was extracted and reverse transcribed with TaqMan microRNA Reverse Transcription kit (Applied Biosystems). Then cDNA was subjected to real-time qPCR analysis on QuantStudio 6 Flex instrument (Applied Biosystems). Relative quantification was achieved by ΔΔC_T_ normalization with the geometric means of housekeeping genes GAPDH and β-actin.

### Data and statistical analysis

Western blots were scanned, and target band densitometry were determined by ImageJ software. The brightness and contrast of western blot images were adjusted just for presentation not for quantification. Statistical analysis was performed with JMP software (SAS Institute). For comparing means of two groups, two tailed student’s t test was applied. For comparison of means across more than two groups with one or two variable(s), analysis of variance (ANOVA) was applied, Tukey’s honest significant difference (HSD) test was followed to compare means between each two groups. Statistical significance threshold was set at 0.05.

### Ethical approval

No Animal work and no human subjects involved in the present work All other procedures were approved and overseen by the Institutional Biosafety Committee (IBC), Office of Research Compliance, Indiana University, Indiana, USA.

## Supplementary Information

Supplementary Information.
