# Supplementary material for: Effects of microRNA-298 on APP and BACE1 translation differ according to cell type and 3′-UTR variation
Source: Sci Rep. 2022 Feb 23;12:3074. doi: 10.1038/s41598-022-05164-4 (PMC8866491; doi:10.1038/s41598-022-05164-4)
Supplement: Supplementary file 1 — Supplementary Information. [file 41598_2022_5164_MOESM1_ESM.pdf]

Effects of microRNA-298 on APP and BACE1 translation differ according to cell type and 3'-UTR variation.

Ruizhi Wang and Debomoy K. Lahiri*

Laboratory of Molecular Neurogenetics, Departments of Psychiatry and of Medical & Molecular Genetics, Indiana Alzheimer Disease Research Center, Indiana University School of Medicine, 320 West 15th Street, Indianapolis, IN-46202, USA

*Corresponding author: dlahiri@iupui.edu

Key words: Astrocytes, dementia, differentiation, neuronal, non-coding RNA, protein expression, post-transcriptional

Legends for supplementary figures

Suppl Fig 1. Effects of miR-298 transfection in fresh media or astrocytic culture media on APP in neurons. A. Neurons transfected with fresh media or transfected with media that had been used to culture astrocytes for 3 days. Densitometric analysis. Each letter represents a different group. **B**. Western blot of APP and β-actin proteins.

Suppl Fig 2. Effects of miR-298 transfection in human microglia cell HMC3. A. Densitometric analysis **B**. CTG. **C**. Western blot of APP, α-tubulin and β-actin proteins.

Suppl Fig 3. Effects of miR-298 transfection in HeLa cells. A. Densitometric analysis **B**. CTG. **C**. Western blot of APP, α-tubulin and β-actin proteins.

Supplement 1

a. APP

**Effect**	**F(df)**	**P**
Treatment	6.86(2)	**0.0061**
Culture media	36.82(1)	< **0.0001**
Treatment*Cultre media	0.91(2)	0.4214

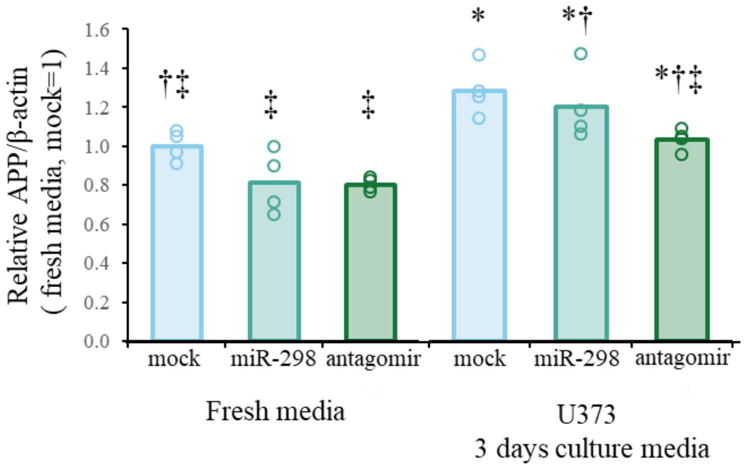

b. western blot

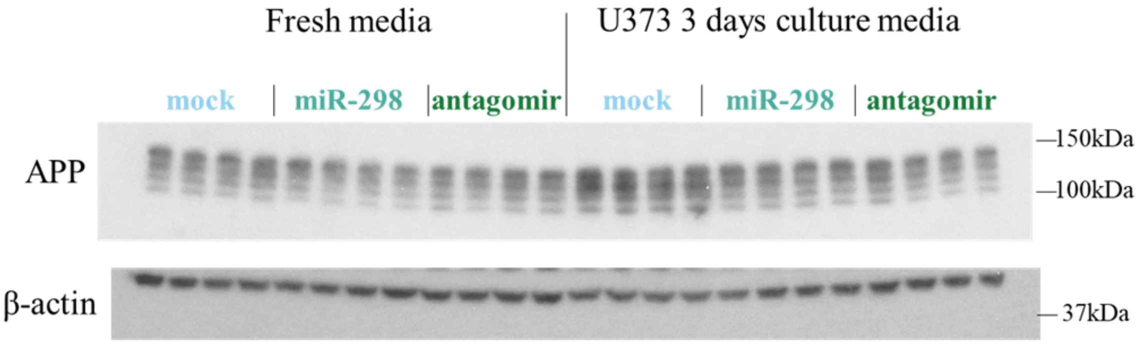

Supplement 2

HMC3

a. APP

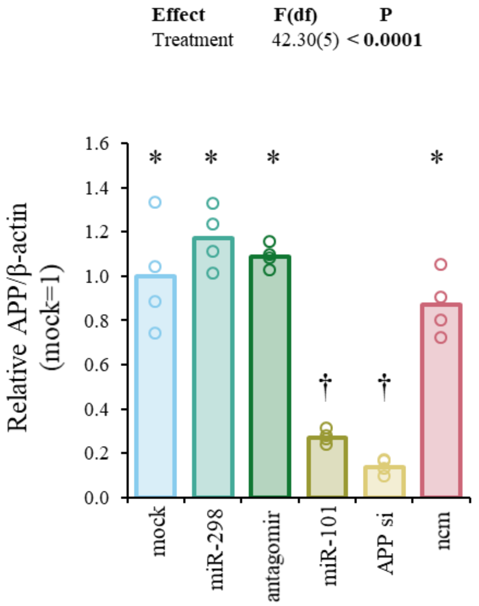

b. CTG

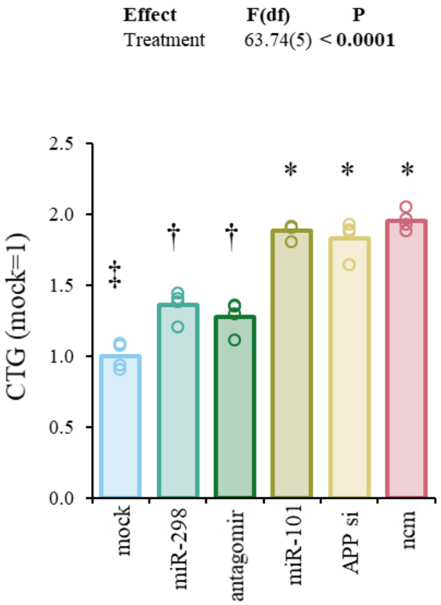

c. western blot

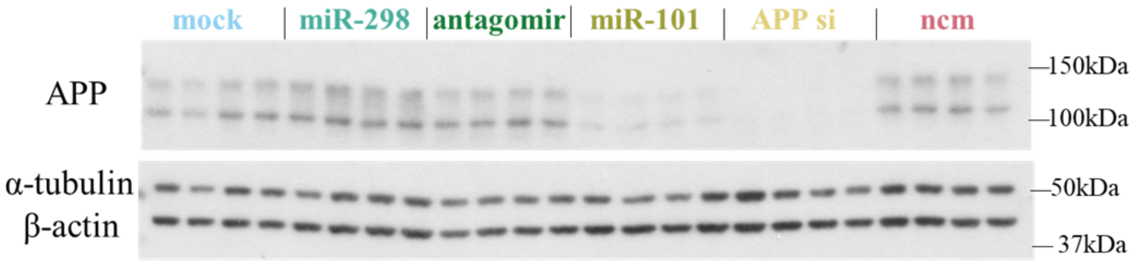

Supplement 3

HeLa

a. APP

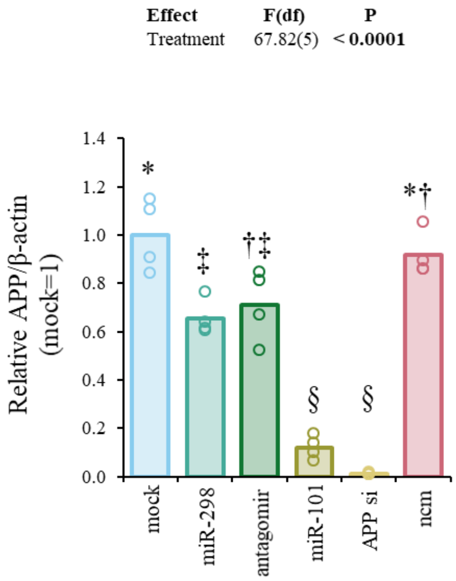

b. CTG

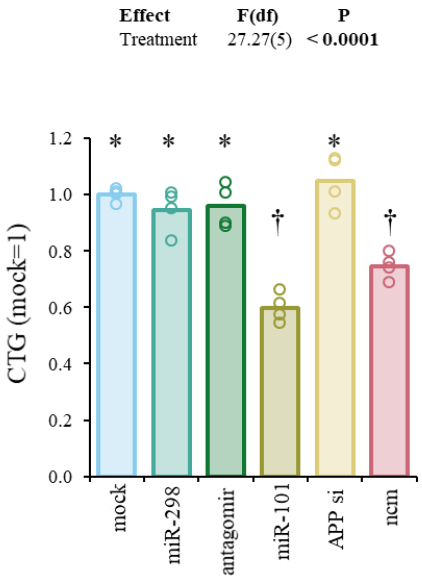

c. western blot

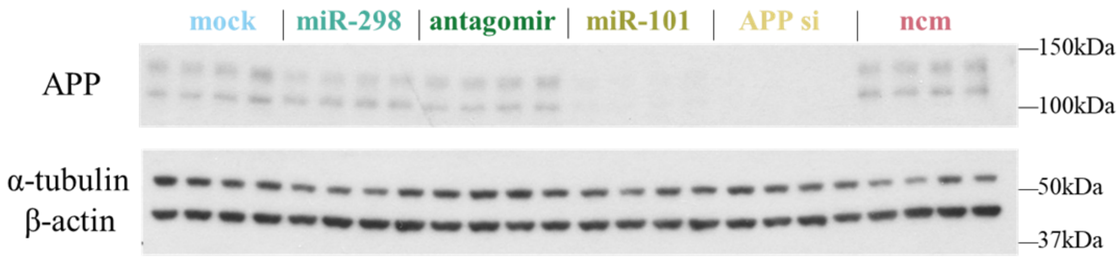

Uncropped western blot images of figures are shown below:

Because the amounts of different proteins and the different antibody dilutions vary, the exposure time of each band needs to be optimized individually. Best images among multiple exposure are applied.

Fig 3f APP blot is shown in red rectangle

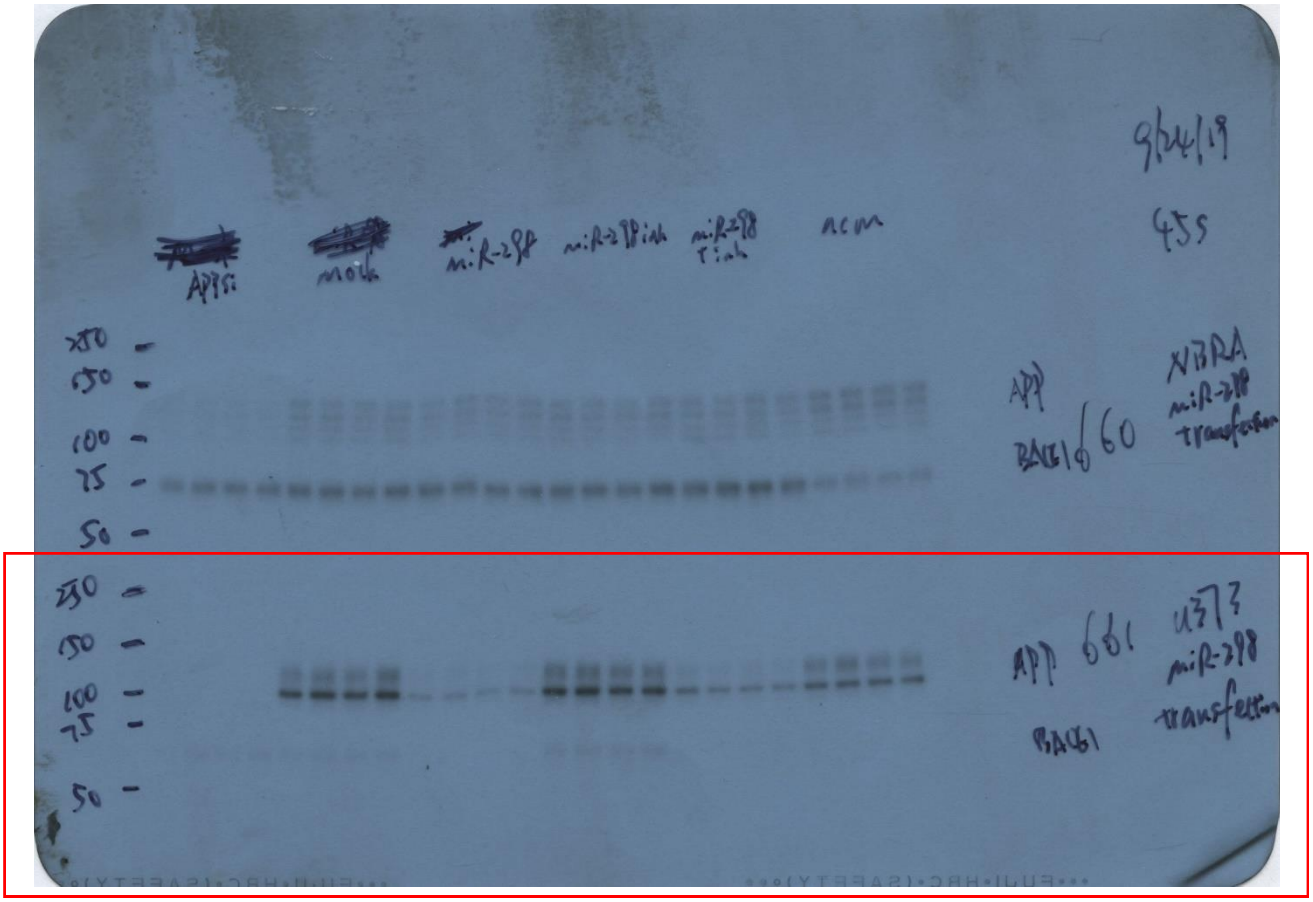

The upper part was SK-N-SH differentiated with ATRA (NBRA) transfected with miR-298, inhibitors and APP, BACE1 siRNA. The lower part in red rectangle are U373 transfected with miR-298, inhibitors and APP, BACE1 siRNA. BACE1 band was too weak to be seen and hence longer exposure with another film was applied.

Fig 3f BACE1 blot is shown in red rectangle.

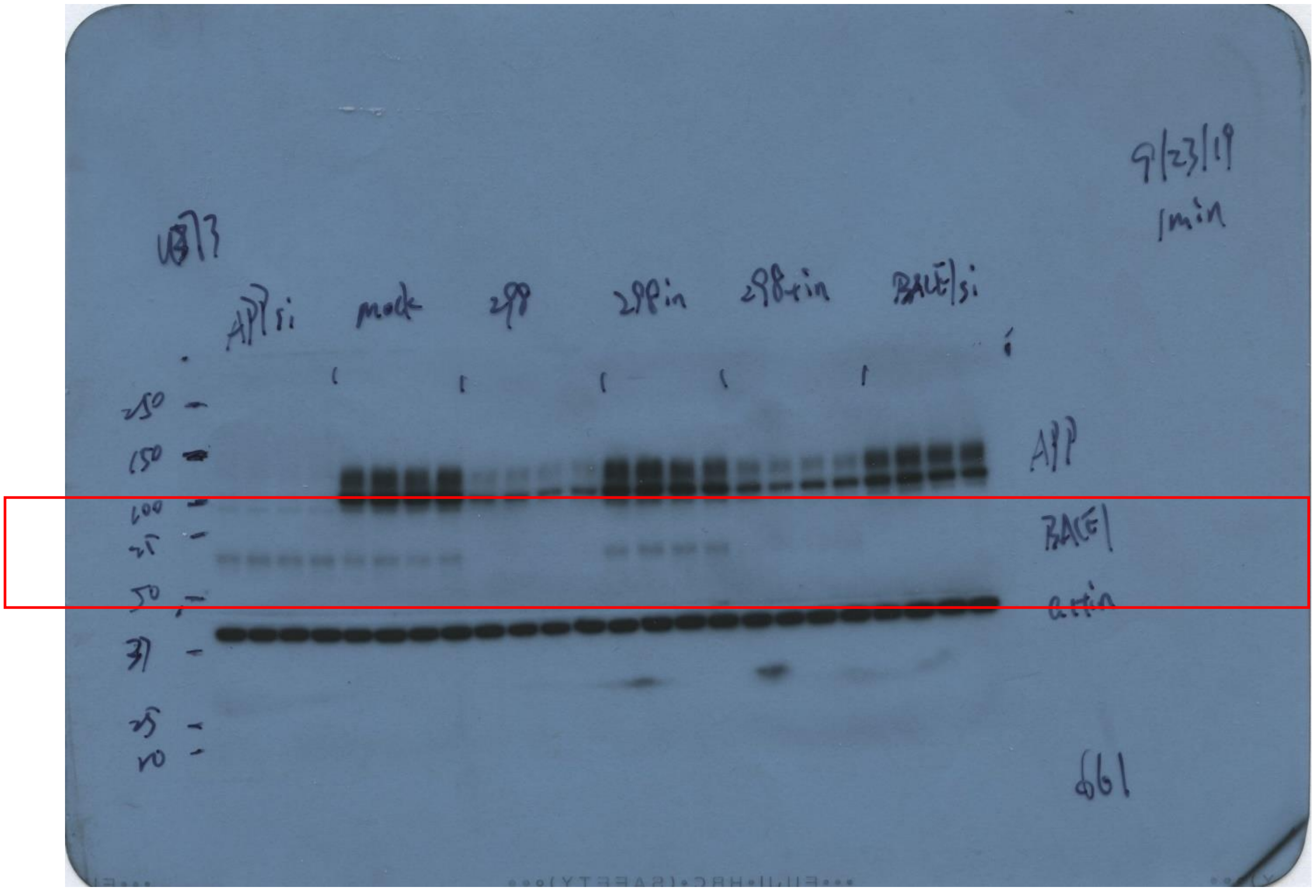

U373 transfected with miR-298, inhibitors and APP, BACE1 siRNA. APP and actin bands were too dark.

Only BACE1 band exposure was appropriate and put into main figures.

Fig 3f. β-actin blot is shown in red rectangle.

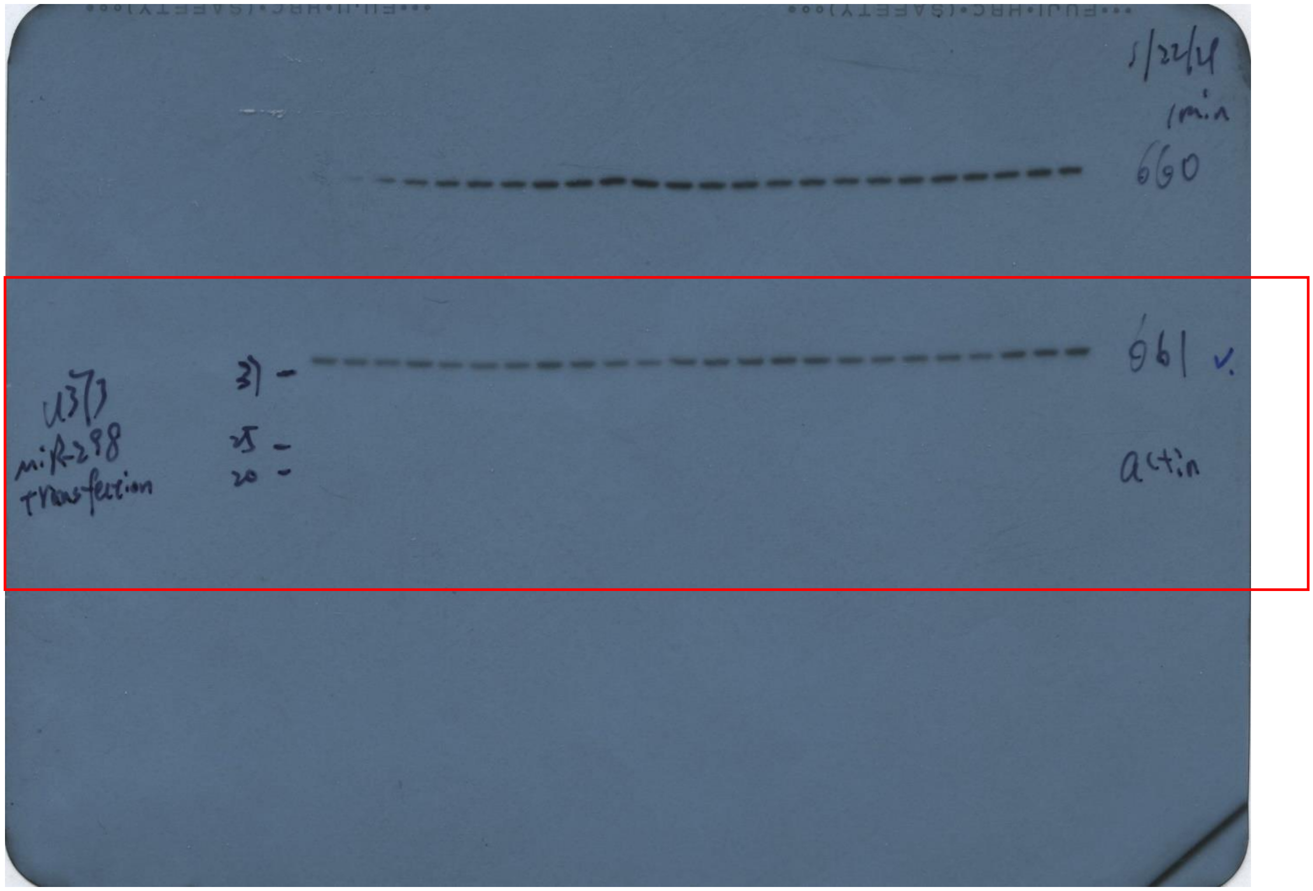

U373 transfected with miR-298, inhibitors and APP, BACE1 siRNA. Actin band showed a reasonable exposure. Upper band was NBRA transfected with miR-298, inhibitors and APP, BACE1 siRNA.

Fig 4e. APP blot.

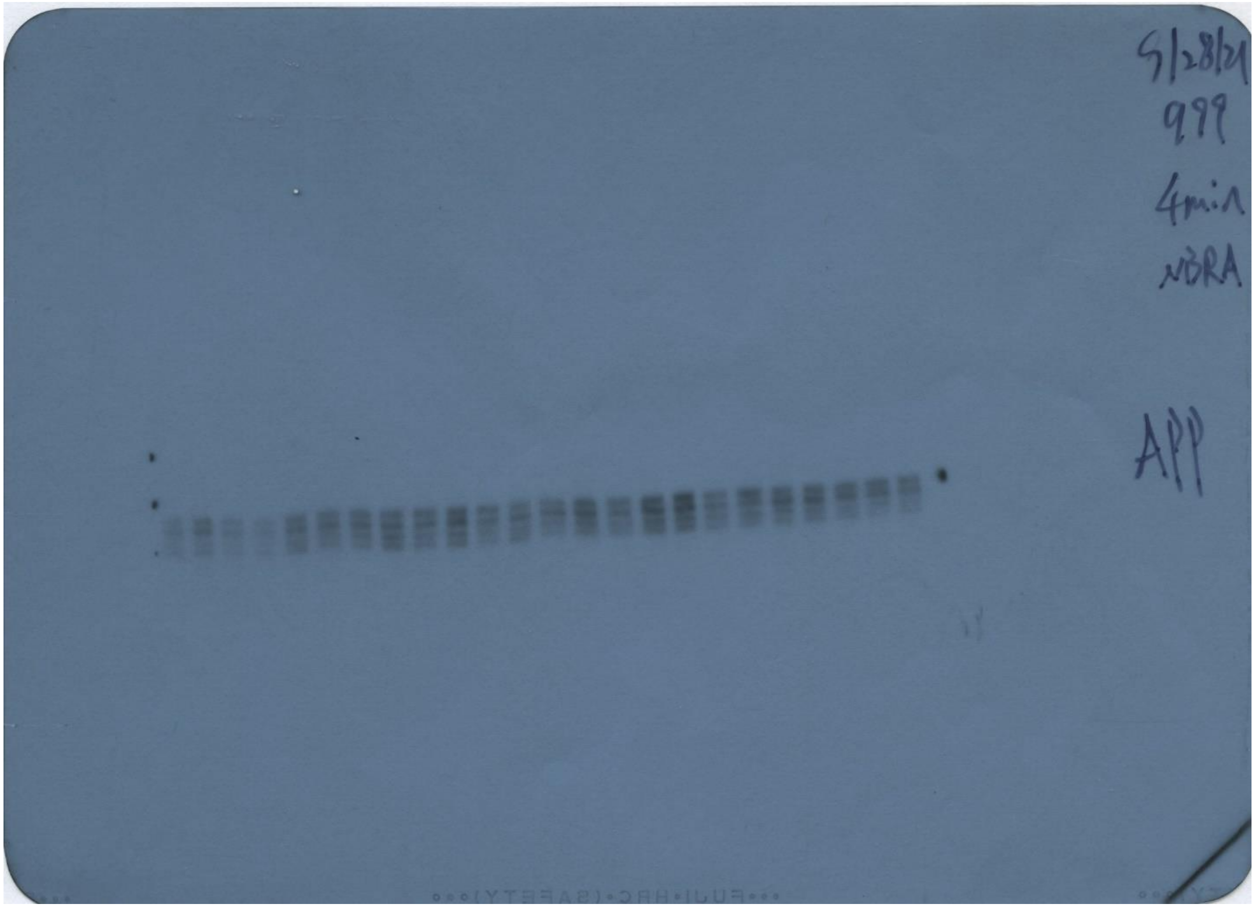

NBRA transfected with miR-298, inhibitors and APP, BACE1 siRNA.

Fig 4e. β-actin blot corresponding to APP blot

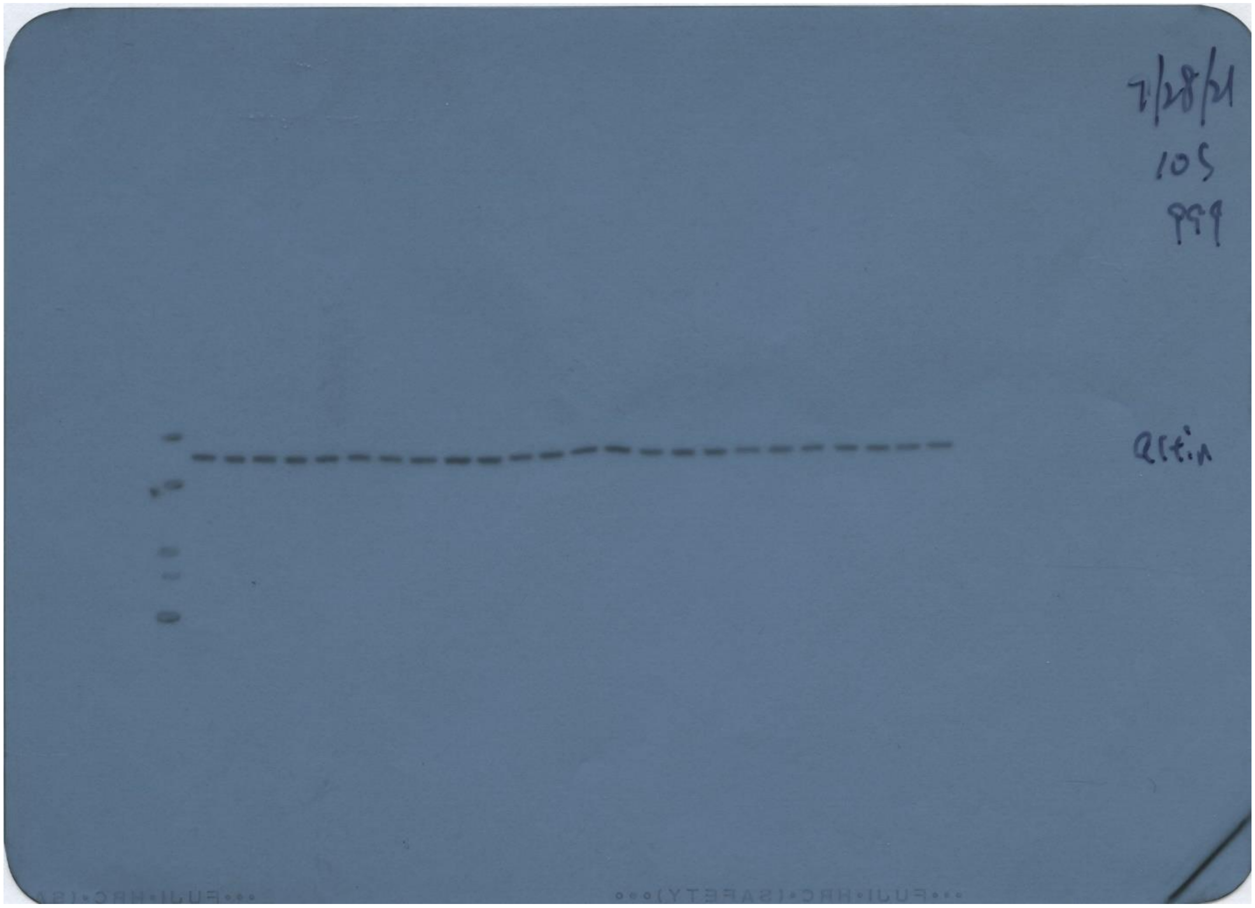

NBRA transfected with miR-298, inhibitors and APP, BACE1 siRNA.

Fig 4e. BACE1 blot is shown in red rectangle.

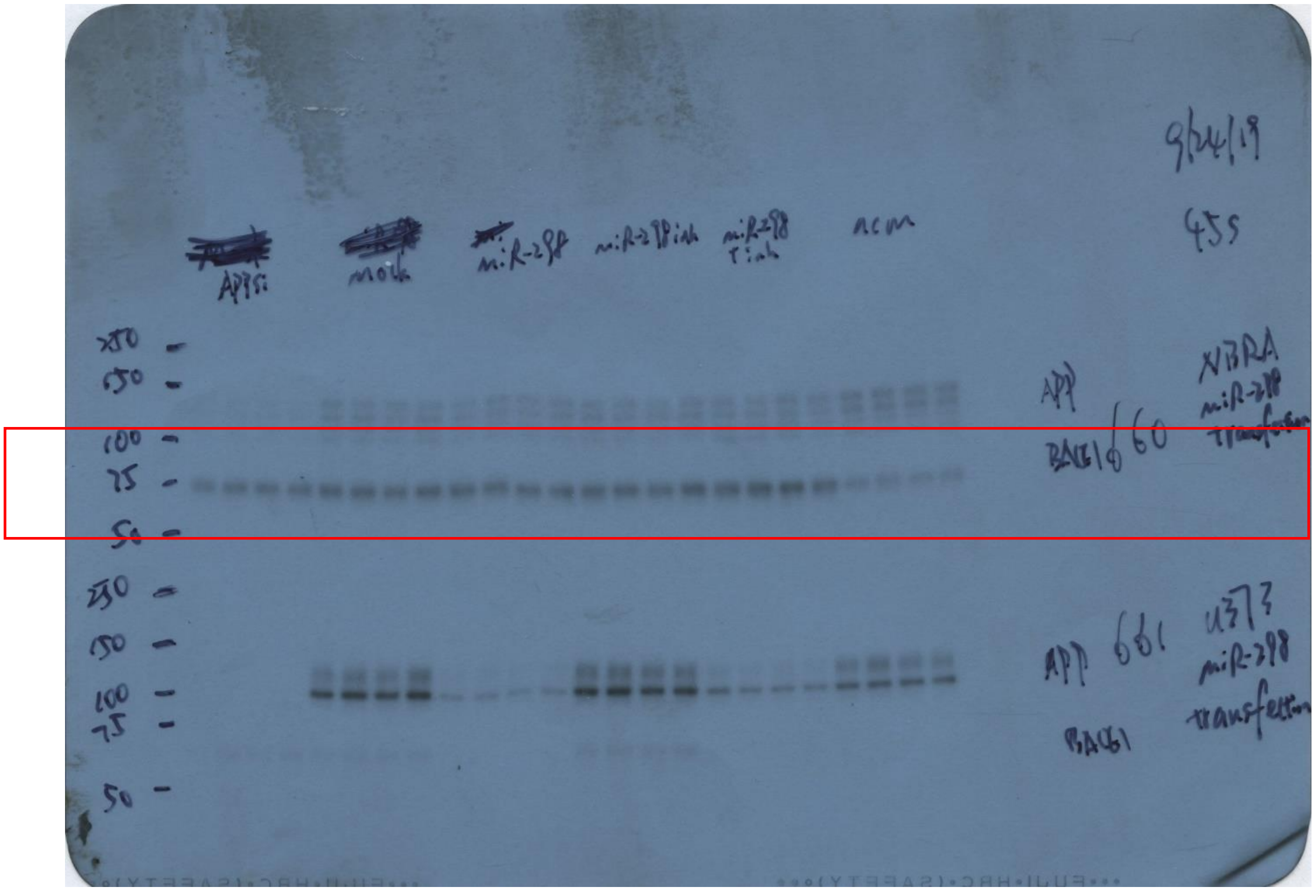

NBRA transfected with miR-298, inhibitors and APP, BACE1 siRNA. Only BACE1 bands was in appropriate exposure time. Lower part was U373 transfected with miR-298, inhibitors and APP, BACE1 siRNA.

Fig 4e. β-actin blot corresponding to BACE1 blot

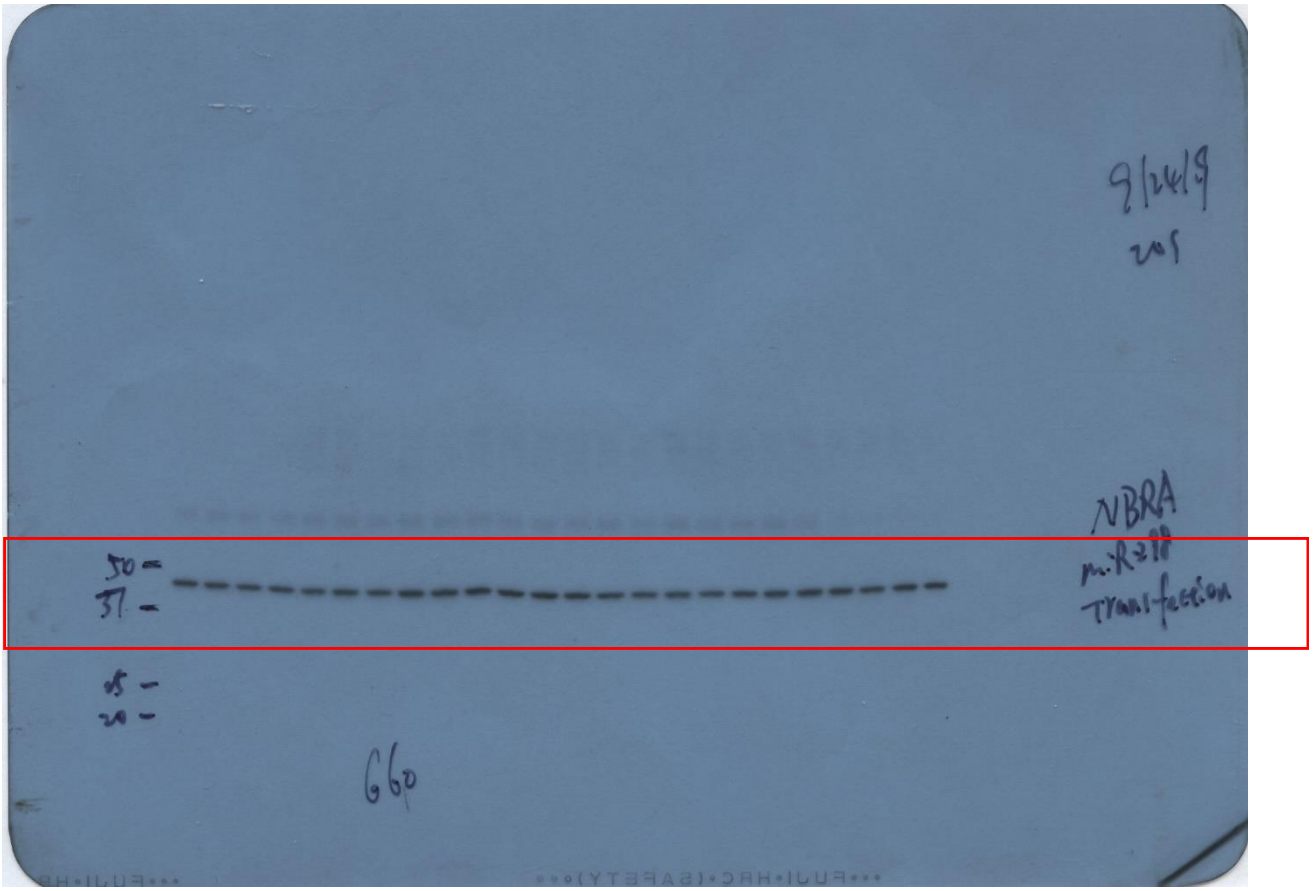

NBRA transfected with miR-298, inhibitors and APP, BACE1 siRNA. Upper bands are APP and BACE1, too light to be seen.

Fig 5d. BACE1 and β-actin blot

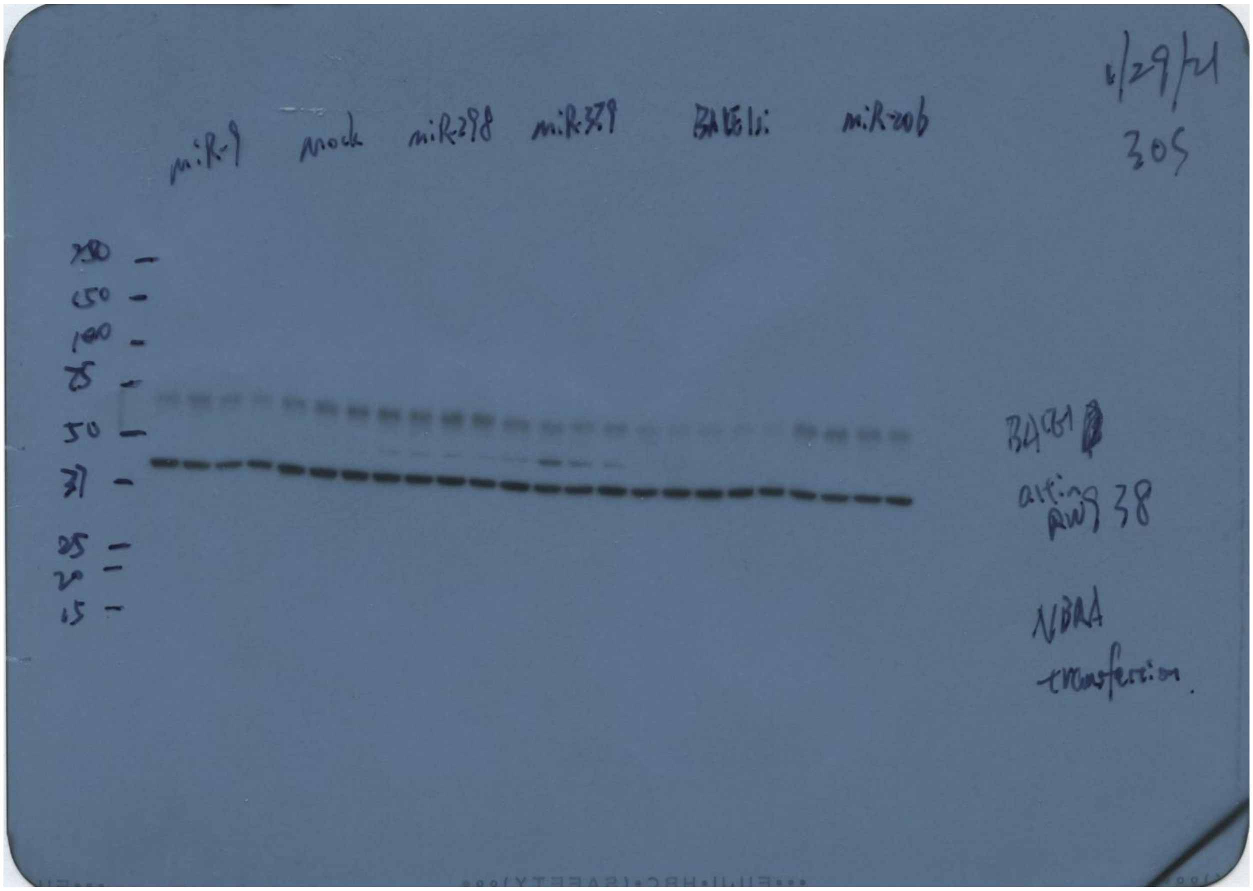

NBRA transfected with miR-9, miR-298, miR-339, miR-20b and BACE1 siRNA. BACE1 and actin bands were shown.

Fig 6d. APP blot

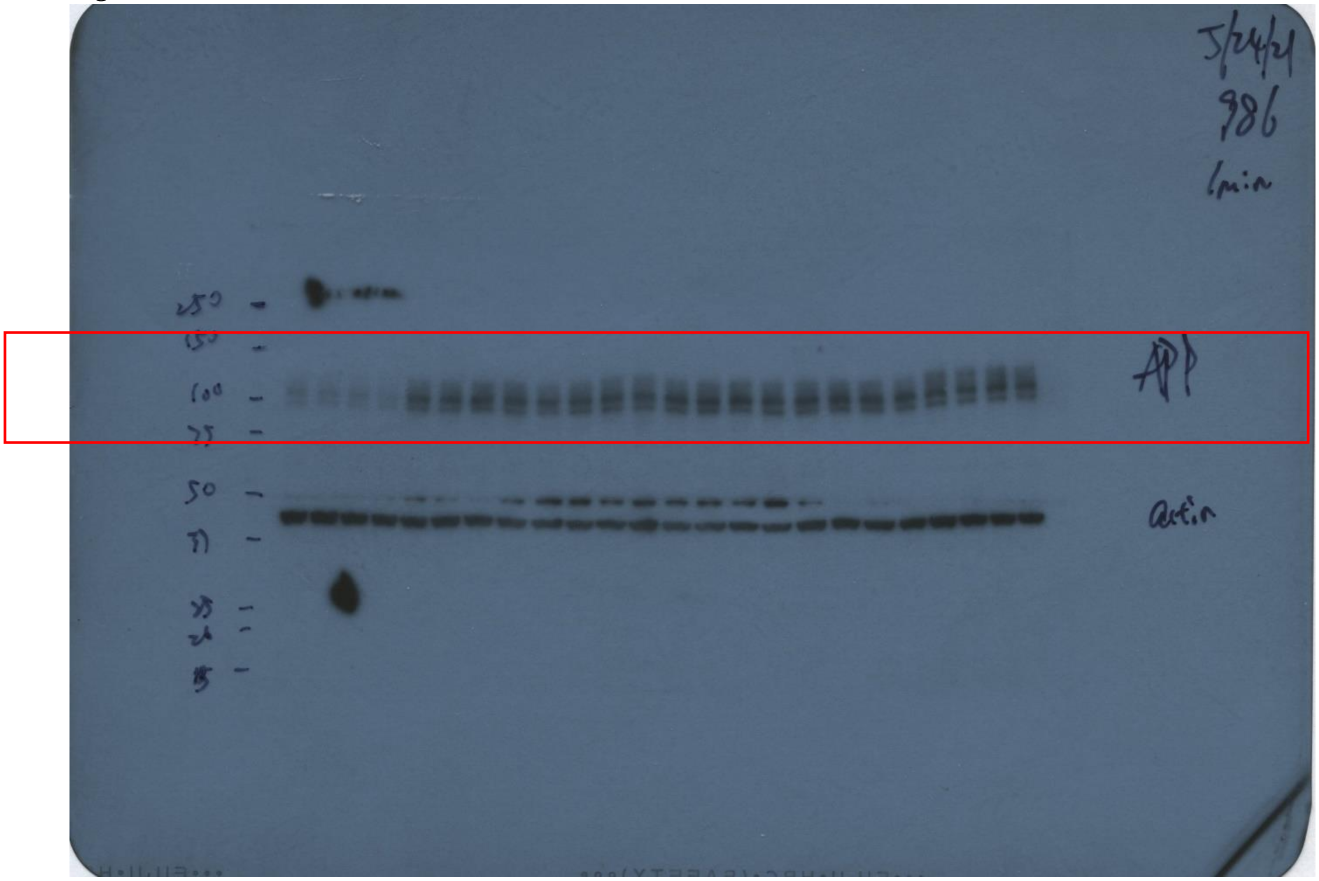

NSC transfected with miR-298, inhibitors, APP siRNA and BACE1 siRNA.

Lower part was actin band, too dark.

Fig 6d. BACE1 blot

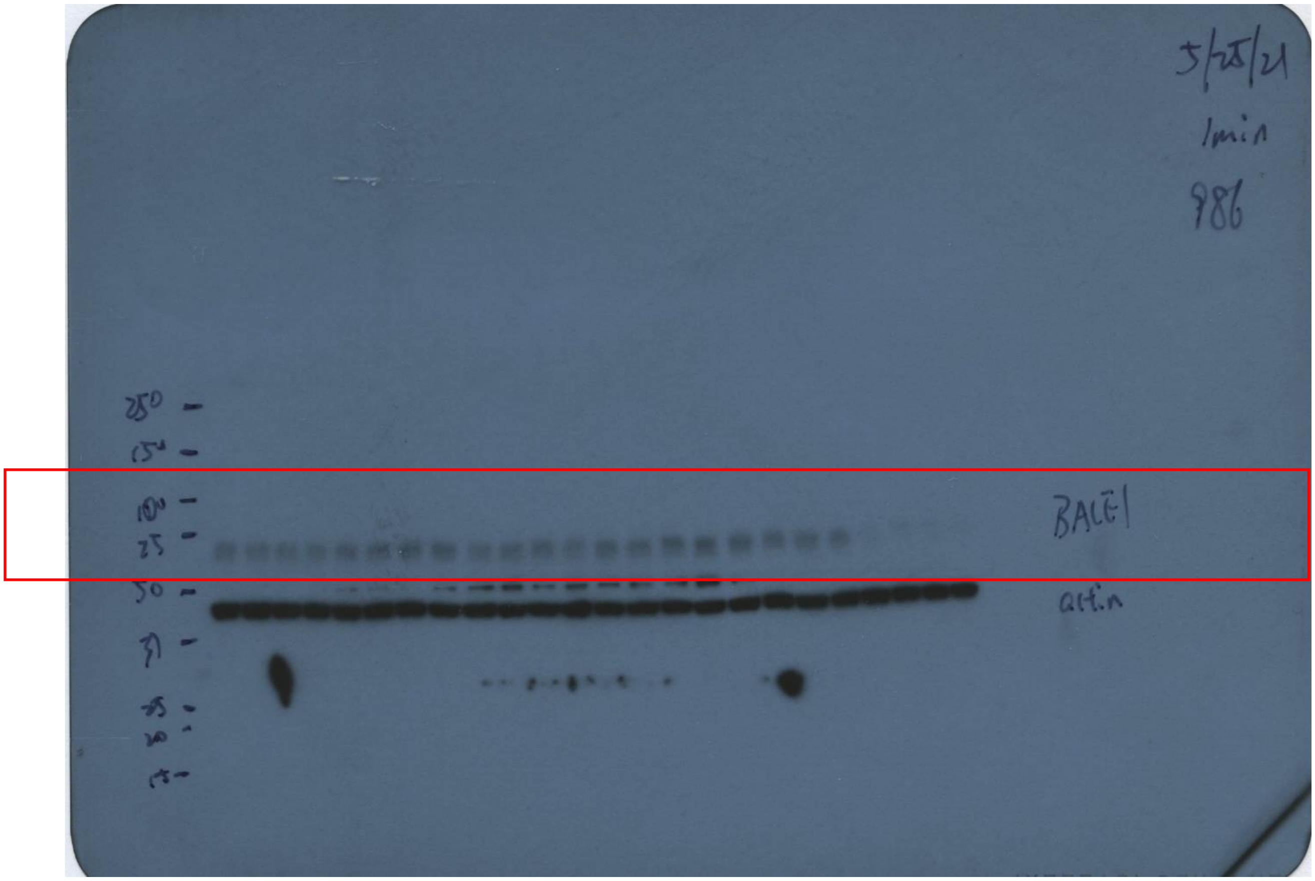

NSC transfected with miR-298, inhibitors, APP siRNA and BACE1 siRNA.

Lower part was actin band, too dark.

Fig 6d. β-actin blot

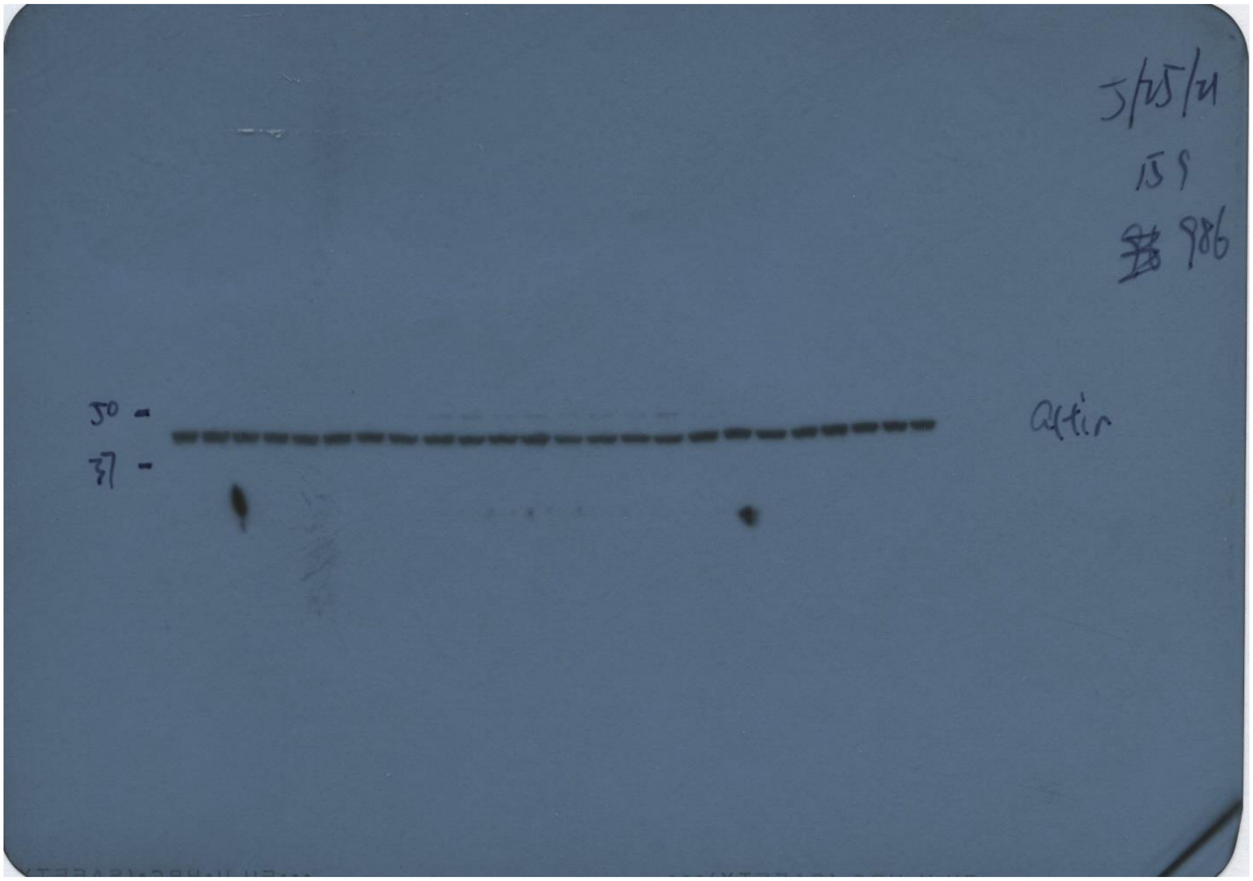

NSC transfected with miR-298, inhibitors, APP siRNA and BACE1 siRNA.

Fig 7b. APP and β-actin blot

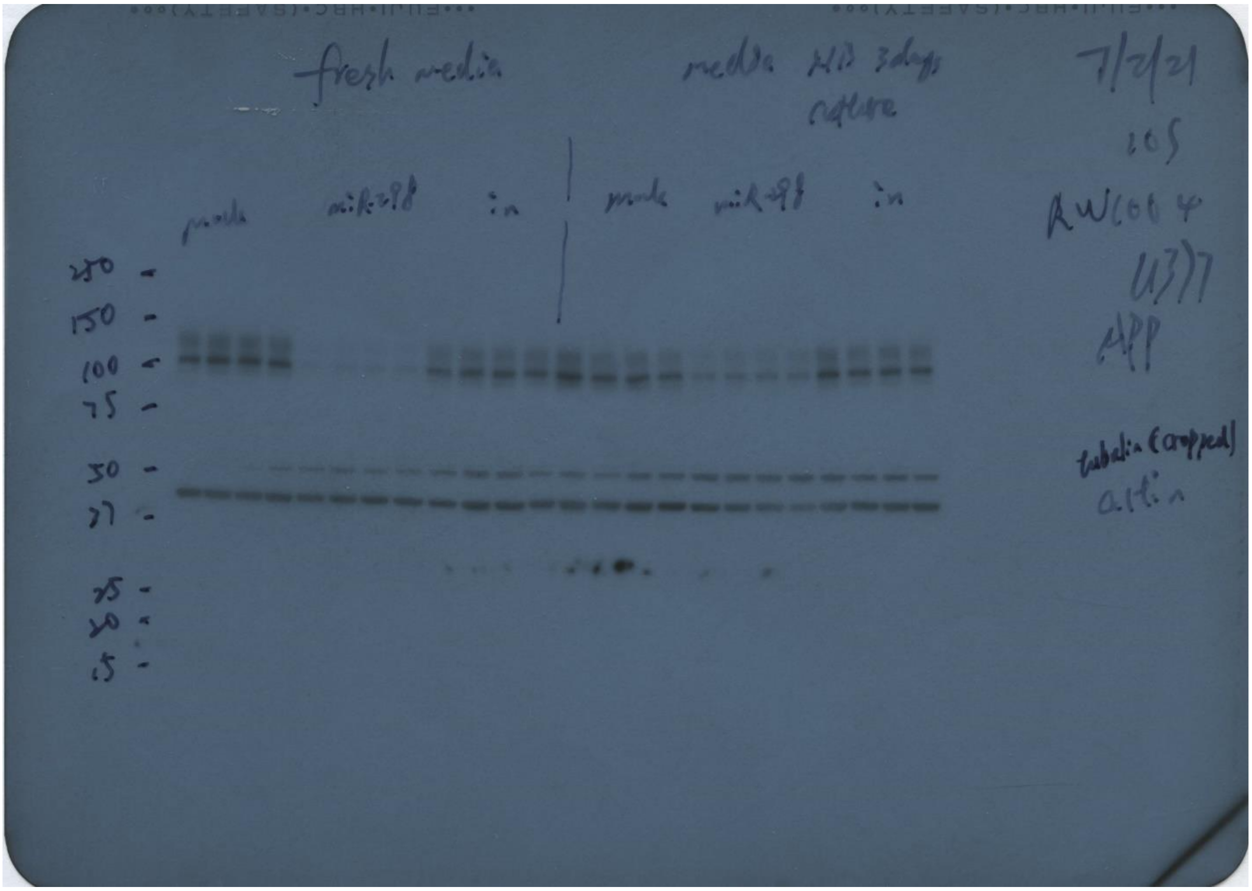

U373 transfected with miR-298 mimics or inhibitors in either fresh opti-MEM media or opti-MEM media from SK-N-SH 3 days culture.

Suppl. fig 1b. APP blot

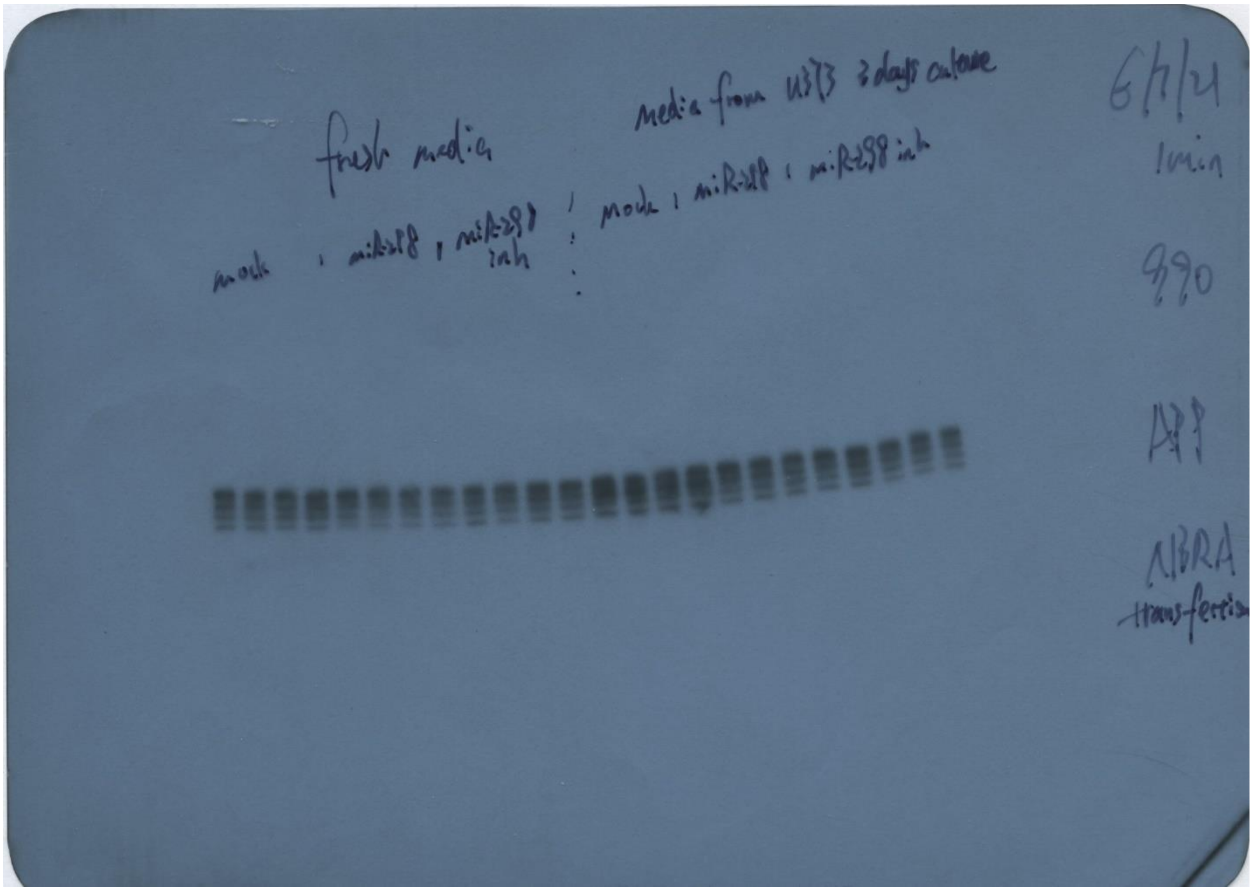

SK-N-SH transfected with miR-298 mimics or inhibitors in either fresh opti-MEM media or opti-MEM media from U373 3 days culture.

Supple fig 1b. β-actin blot

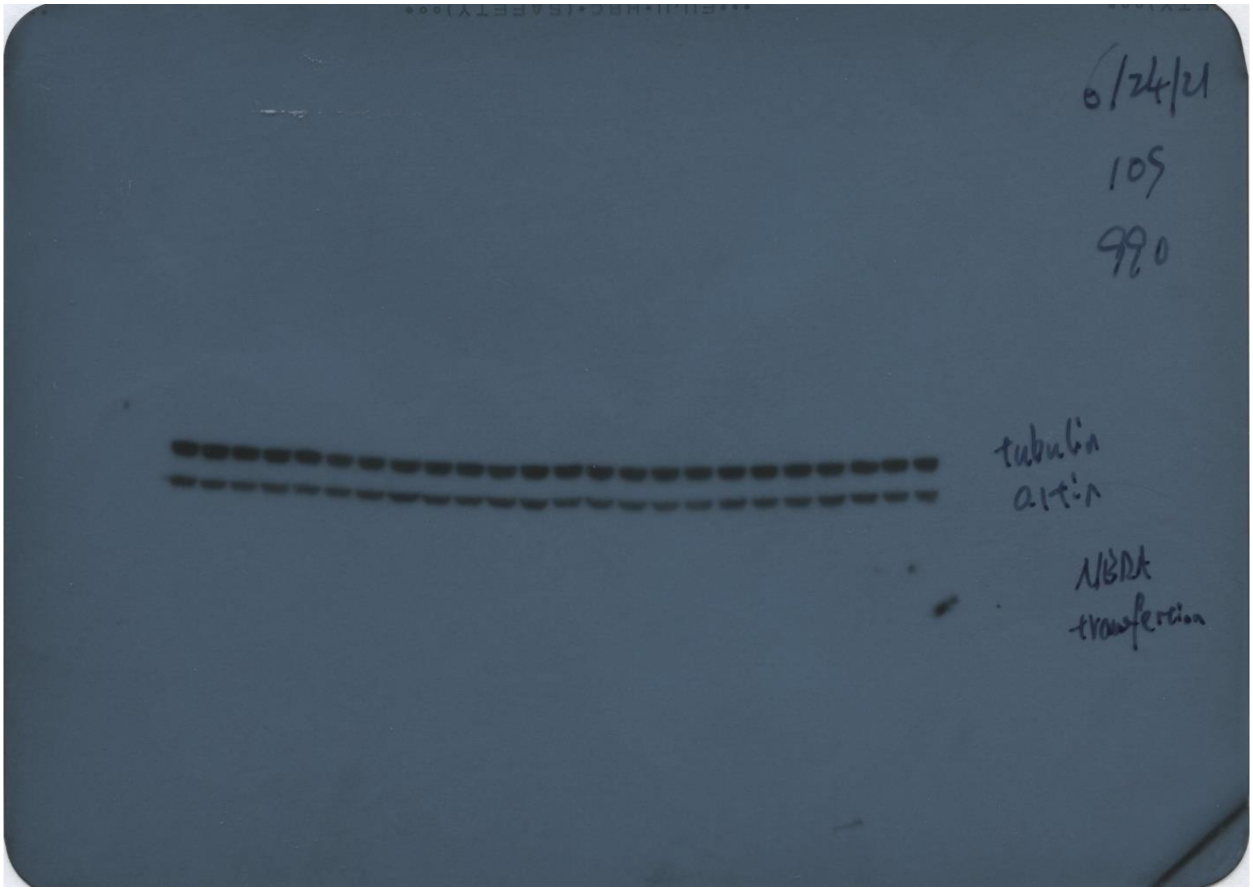

SK-N-SH transfected with miR-298 mimics or inhibitors in either fresh opti-MEM media or opti-MEM media from U373 3 days culture.

Supple fig 2c. APP and β-actin blot

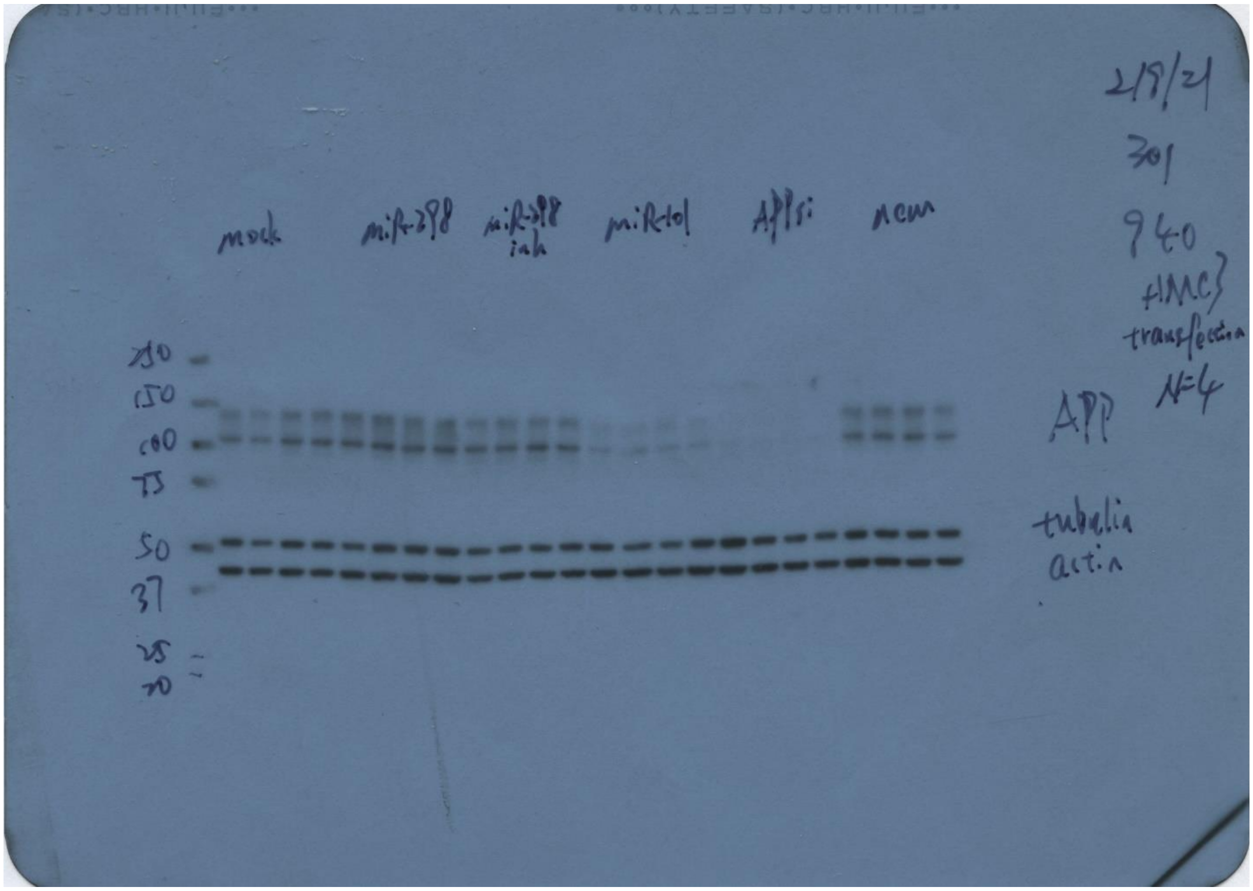

HMC3 transfected with miR-101, miR-298 and APP siRNA. APP, actin and tubulin blots.

Supple fig 3c. APP and β-actin blot

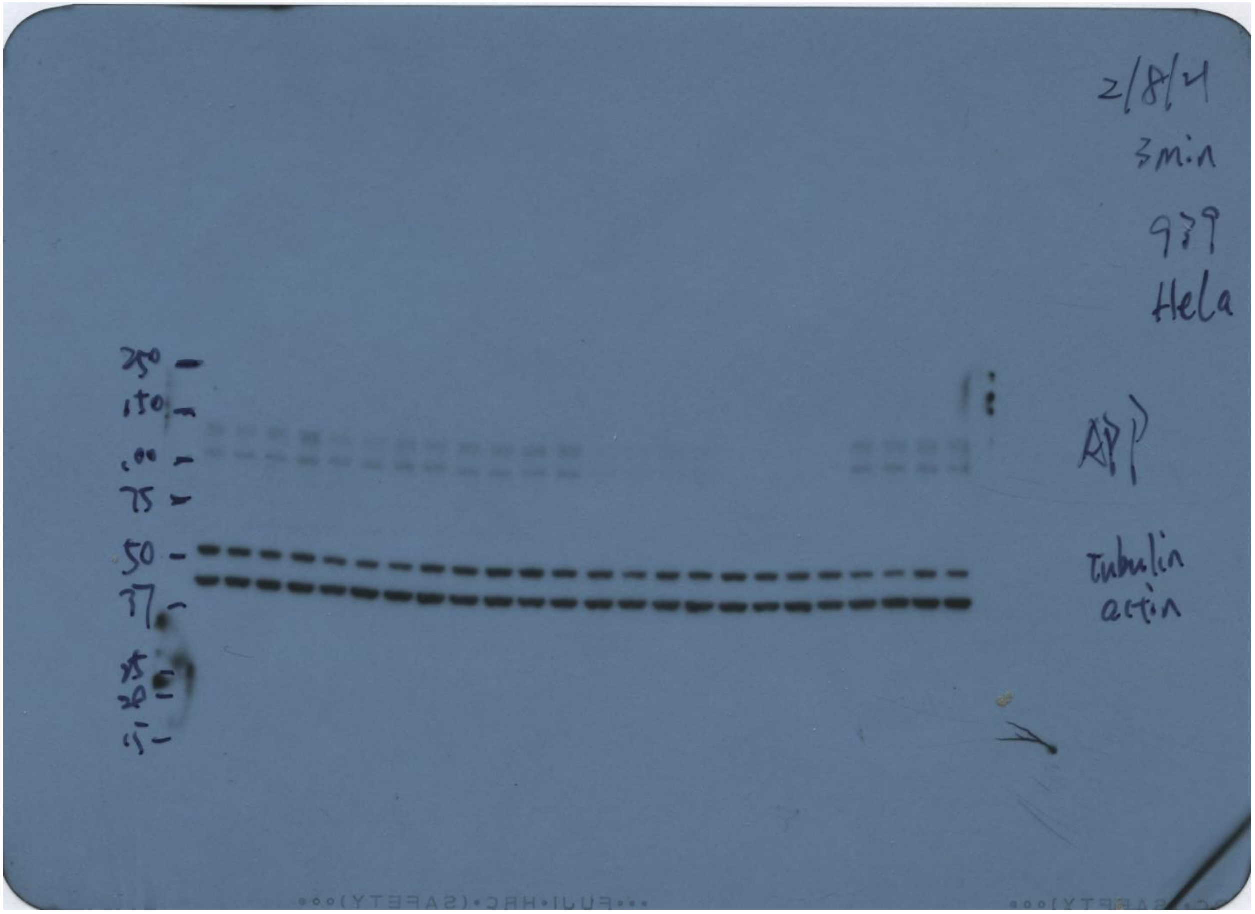

HeLa transfected with miR-101, miR-298 and APP siRNA. APP, actin and tubulin blots.
